# Alternative assembly of respiratory complex II connects energy stress to metabolic checkpoints

**DOI:** 10.1038/s41467-018-04603-z

**Published:** 2018-06-07

**Authors:** Ayenachew Bezawork-Geleta, He Wen, LanFeng Dong, Bing Yan, Jelena Vider, Stepana Boukalova, Linda Krobova, Katerina Vanova, Renata Zobalova, Margarita Sobol, Pavel Hozak, Silvia Magalhaes Novais, Veronika Caisova, Pavel Abaffy, Ravindra Naraine, Ying Pang, Thiri Zaw, Ping Zhang, Radek Sindelka, Mikael Kubista, Steven Zuryn, Mark P. Molloy, Michael V. Berridge, Karel Pacak, Jakub Rohlena, Sunghyouk Park, Jiri Neuzil

**Affiliations:** 1 School of Medical Sciences, Griffith University, Southport, 4222 Qld Australia; 2 Clem Jones Centre for Ageing Dementia Research, Queensland Brain Institute, University of Queensland, Brisbane, 4072 Qld Australia; 3 Department of Biochemistry and Molecular Biology, Shenzhen University School of Medicine, Shenzhen, 518060 China; 4 College of Pharmacy, Natural Product Research Institute, Seoul National University, Seoul, 08826 Korea; 5 Institute of Biotechnology, Czech Academy of Sciences, Prague-West, 25250 Czech Republic; 6 Institute of Molecular Genetics, Czech Academy of Sciences, Prague, 14220 Czech Republic; 7 Eunice Kennedy Shriver National Institute of Child Health and Human Development, National Institutes of Health, Bethesda, 20892 MD USA; 8 Faculty of Science, University of South Bohemia, Ceske Budejovice, 37005 Czech Republic; 9 Australian Proteome Analysis Facility, Macquarie University, North Ryde, 2109 NSW Australia; 10 TATAA Biocenter, Gothenburg, 41103 Sweden; 11 Malaghan Institute of Medical Research, Wellington, 6242 New Zealand

## Abstract

Cell growth and survival depend on a delicate balance between energy production and synthesis of metabolites. Here, we provide evidence that an alternative mitochondrial complex II (CII) assembly, designated as CII_low_, serves as a checkpoint for metabolite biosynthesis under bioenergetic stress, with cells suppressing their energy utilization by modulating DNA synthesis and cell cycle progression. Depletion of CII_low_ leads to an imbalance in energy utilization and metabolite synthesis, as evidenced by recovery of the de novo pyrimidine pathway and unlocking cell cycle arrest from the S-phase. In vitro experiments are further corroborated by analysis of paraganglioma tissues from patients with sporadic, SDHA and SDHB mutations. These findings suggest that CII_low_ is a core complex inside mitochondria that provides homeostatic control of cellular metabolism depending on the availability of energy.

## Abstract (web summary)

Mitochondrial complex II is normally composed of four subunits. Here the authors show that bioenergetic stress conditions give rise to a partially assembled variant of complex II, which shifts the anabolic pathways to less energy demanding processes.

## Introduction

Mitochondria are semi-autonomous organelles found in the majority of eukaryotic cells. They have their own genome (mitochondrial DNA, mtDNA), which encodes subunits of respiratory complexes and RNA components for mitochondrial protein synthesis. Major roles of mitochondria include generation of energy and synthesis of metabolites. Molecules that are a source of energy are oxidized in a series of biochemical reactions within the tricarboxylic acid (TCA) cycle. Intermediate products of the TCA cycle are used as signaling molecules and as building blocks for various macromolecules, while NADH and FADH_2_ are metabolized via oxidative phosphorylation (OXPHOS) to yield ATP. OXPHOS comprises five complexes, CI–CV. CII (succinate dehydrogenase, SDH) contains nuclear-encoded SDHA, SDHB, SDHC, and SDHD subunits, which are recognized as tumor suppressors^1–3^. Besides its role in OXPHOS, CII converts succinate to fumarate in the TCA cycle, and is thus at the crossroad of the TCA cycle and OXPHOS^4^.

Clinical data document the presence of somatic mutations in mtDNA in cancer in both the regulatory D-LOOP and the coding regions^5–8^. Previous research has mainly concerned the effects of mtDNA mutations on CI, CIII, CIV, and CV, with CII having been a relatively minor focus. This is explained by the fact that unlike all other OXPHOS complexes, CII does not contain mtDNA-encoded subunits, and therefore no direct effect of mtDNA defects on CII assembly and function has been expected. Further, individual respiratory complexes form supercomplexes (SCs)^9–11^, while CII acts as a stand-alone complex, with only one report indicating that it can be an SC component^12^.

This study investigates whether CII subunits and mtDNA could have any form of interaction in energy production, and if so, whether there is a functional relation that provides an advantage to cells with mtDNA mutations. We show that depletion of mtDNA has an unexpected effect on CII assembly, causing a shift from its tetrameric, fully processed, and assembled form to a slower migrating complex of ~100 kDa, referred to here as complex II_low_ (Cll_low_). Our data suggest that CII_low_ links bioenergetic stress to negative regulation of de novo pyrimidine synthesis and cell cycle progression, which is supported by clinical data from paraganglioma patients with mutations in SDH subunits, indicating that Cll_low_ plays an important role in homeostatic control of metabolite synthesis under bioenergetic stress.

## Results

### mtDNA-linked bioenergetics defects affect the assembly of CII

**Fig. 1:**
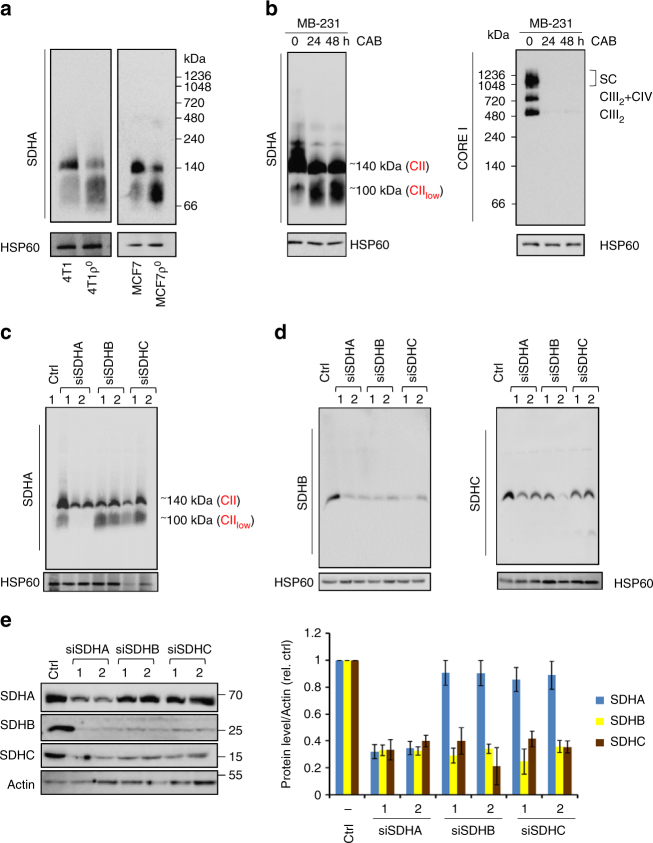
mtDNA dysfunction affects CII assembly. **a** NBGE analyses of CII assembly from digitonin-solubilized mitochondria isolated from 4T1 and MCF7 cells and their ρ^0^ counterparts. **b** NBGE showing formation of CII_low_ and depletion of CIII assembly upon suppression of expression of mtDNA-encoded genes with CAB at indicated time points. **c**, **d** NBGE of CII using mitochondria isolated from 4T1 cells transfected with siRNA against SDHA, SDHB, or SDHC. **e** WB after SDS-PAGE of steady-state levels of CII subunits in 4T1 cells treated with siRNAs as shown (left panel). Right panel shows quantification of WB in the left panel related to actin. The numbers ‘1’ and ‘2’ in **c**–**e** refer to two different siRNAs. Data shown are mean values ± SD; images are representative of three independent experiments

Unlike other respiratory complexes, CII is encoded by nuclear DNA and is genetically independent of mtDNA. To understand if mtDNA dysfunction affects CII indirectly, we tested the effect of mtDNA perturbation^8,13–15^ on CII assembly. Native blue gel electrophoresis (NBGE) of mitochondria isolated from murine 4T1 and human MCF7 cells without mtDNA (ρ^0^ cells) revealed that CII exists in two hetero-oligomeric forms of ~100 kDa and 124 kDa (migrating on NBGE at ~140 kDa). In 4T1ρ^0^ and MCF7ρ^0^ cells, SDHA was mainly present as CII_low_, with no known biological function reported to date, and to a lesser degree within fully assembled CII (Fig. 1a). The finding of predominant CII_low_ in 4T1ρ^0^ and MCF7ρ^0^ cells suggests that this form of CII may have a role in (patho)physiological situations where mtDNA is damaged. The size of the processed SDHA protein is ~69 kDa, while CII_low_ migrates on native gels at ~100 kDa. Hence, additional protein components beyond SDHA must be present in CII_low_.

We next examined CII assembly in human MDA-MB-231 (MDA231) cells. Inhibition of mitochondrial protein synthesis with chloramphenicol (CAB), a blocker of mitochondrial translation^16–18^, resulted in depletion of CIII and SCs (Fig. 1b, right panels). Similarly, as with 4T1ρ^0^ and MCFρ^0^ cells, CAB-treated MDA231 cells accumulated CII_low_ containing SDHA (Fig. 1b, left panels), suggesting that this form of CII may have (patho)physiological relevance when expression of mtDNA-encoded proteins is compromised.

### SDHA is a stable constituent of CII_low_

To better characterize the subunit composition of CII_low_, SDHA, SDHB, and SDHC subunits were knocked down one at a time in 4T1 cells using two different siRNAs (siRNA1 and siRNA2), and CII assembly state was followed by NBGE. Depletion of any of the three subunits reduced the level of CII. Interestingly, only the knockdown of SDHA, but not of the other CII subunits, resulted in marked reduction of CII_low_ (Fig. 1c and d), corroborating the presence of SDHA, but not SDHB or SDHC, in CII_low_.

The identification of SDHA in CII_low_ suggests that it may be more stable than the other CII subunits. We therefore examined the steady state of each subunit in our knockdown cell lines. Depletion of SDHB led to a decrease in the steady state of SDHC, and knockdown of SDHC led to a decrease in the steady state of SDHB. Interestingly, in SDHA knockdown cells, both SDHB and SDHC levels decreased by ~70%. Knocking down either SDHB or SDHC had no effect on the steady state of SDHA (Fig. 1e). It is likely that depletion of SDHA leads to low levels of SDHB and SDHC, probably due to low stability of unassembled subunits as indicated previously^19–21^.

### Characterization of the CII_low_ form of SDH

**Fig. 2:**
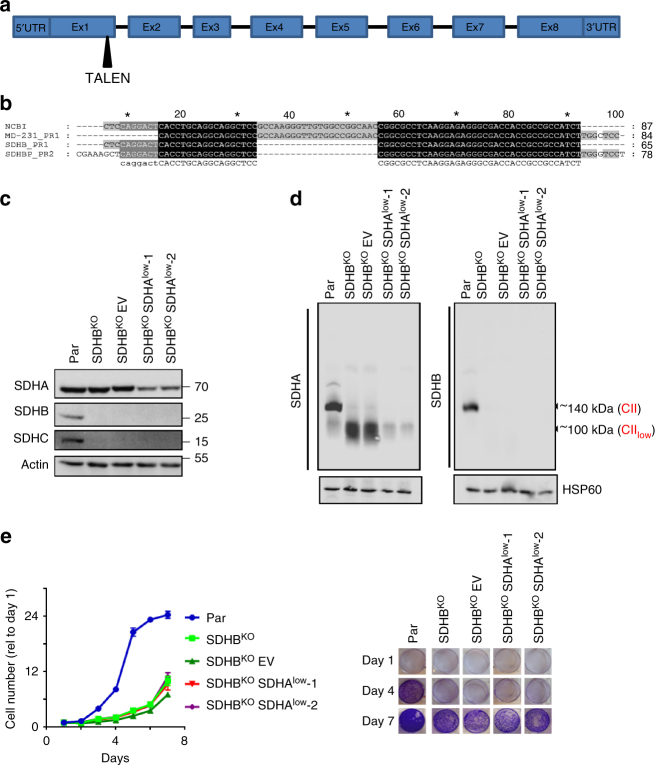
SDHB depletion stabilizes CII_low_. **a** A scheme showing the structure of the *SDHB* gene and exon1 as the TALEN target. **b** DNA sequence alignment of the *SDHB* exon 1 from the GenBank nucleotide sequence database, and parental and SDHB^KO^ MDA231 cells. Sanger sequencing was done from the PCR product amplified using two pairs of primers (PR1 and PR2) using genomic DNA as a template. **c** WB after SDS-PAGE of CII subunits in individual sublines, as shown. SDHB^KO^EV cells are SDHB^KO^ cells transfected with empty vector and were used as a control for stable shRNA transfections. **d** NBGE of mitochondria isolated from parental, SDHB^KO^, SDHB^KO^EV, and SDHB^KO^SDHA^low^ cells showing three variants of SDHA, using anti-SDHA IgG and anti-SDHB IgG. **e** Proliferation of MDA231 subline was analyzed at the indicated time points using the crystal violet method. Panel on the right shows crystal violet staining at 1, 4, and 7 days. Data are normalized to day 1. Data shown are mean values ± SD (*n* = 3); images are representative of three independent experiments

Based on crystal structure of CII^22^ (Supplementary Fig. 1a) and our data in Fig. 1e, we hypothesized that depletion of SDHB will result in cells containing only CII_low_. We thus generated SDHB knockout (KO) MDA231 cells targeting exon 1 (Fig. 2a and b). As with 4T1 cells (Fig. 1c and e), the steady-state level of SDHA was unchanged in SDHB^KO^ MDA231 cells (Fig. 2c). In order to deplete CII_low_, SDHB^KO^ MDA231 cells were transfected with two different *SDHA* shRNAs (SDHB^KO^SDHA^low^-1 and SDHB^KO^SDHA^low^-2) and an empty vector (SDHB^KO^EV) as a control (Fig. 2c; Supplementary Fig. 1b). NBGE analysis showed high levels of CII_low_ in SDHB^KO^ MDA231 cells and little CII_low_ in SDHB^KO^SDHA^low^ cells (Fig. 2d). We thus prepared models of cells with three variants of CII assembly: fully assembled CII and low levels of CII_low_ (parental cells), cells with only CII_low_ (SDHB^KO^ SDHB^KO^EV cells), and cells lacking both CII and CII_low_ (SDHB^KO^SDHA^low^ cells). These models showed low proliferation for both SDHB^KO^ and SDHB^KO^SDHA^low^ cells (Fig. 2e).

To specify its molecular composition, we transfected SDHB^KO^ cells with SDHA-FLAG and immunoprecipitated the cell-free extract using anti-FLAG IgG. The immunoprecipitate was subjected to tryptic digest followed by MS analysis (Supplementary Fig. 2a), with results of the screen in Supplementary Fig. 2a and Supplementary Data 1. The analysis revealed the presence of the CII assembly factors SDHAF2 (16.7 kDa) and SDHAF4 (9.9 kDa). While SDHAF2 was readily detectable on NBGE membranes in CII_low_ (Supplementary Fig. 2b), the available antibodies did not allow reproducible detection of SDHAF4 after NBGE, and its presence was verified by other means (see below).

We next re-expressed SDHB-FLAG in SDHB^KO^ cells to see whether this would rescue the parental phenotype and whether CII_low_ is reversible. SDHB-reconstituted (SDHB^rec^) cells were assessed by Western blotting (WB) following SDS-PAGE for the presence of SDHA, SDHB, SDHAF2, and SDHAF4. Supplementary Fig. 2c documents similar levels of SDHA in all three sublines and high levels of SDHB in parental and SDHB^rec^ cells. In contrast, both SDHAF2 and SDHAF4 were low in parental and SHDB^rec^ cells, but accumulated in SDHB^KO^ cells. We then tested the three sublines by NBGE followed by WB using anti-SDHA IgG and anti-SDHAF2 IgG. Supplementary Fig. 2d reveals that mature CII is re-assembled and the amount of CII_low_ reverts to parental levels in SDHB^rec^ cells, indicating SDHB-dependent reversibility of alternatively assembled CII. Furthermore, SDHAF2 and SDHAF4 were detectable only in SDHB^KO^ cells that lack mature CII but feature CII_low_, supporting the presence of these assembly factors in CII_low_ cells.

To verify the reversibility of CII_low_ in response to stress, we treated parental cells with CAB for 48 h, followed by 24 h recovery. The amount of CII_low_ was evaluated by NBGE using antibodies against SDHA and SDHAF2, two CII_low_ components readily detectable in this assay (Supplementary Fig. 2e). Treatment with CAB increased the level of CII_low_, which reverted to baseline within 24 h of treatment cessation. Together with the SDHB^rec^ data, this indicates reversibility of CII_low_ assembly, providing flexibility in response to mitochondrial bioenergetic stress.

### CII_low_ does not modulate mitochondrial bioenergetics

**Fig. 3:**
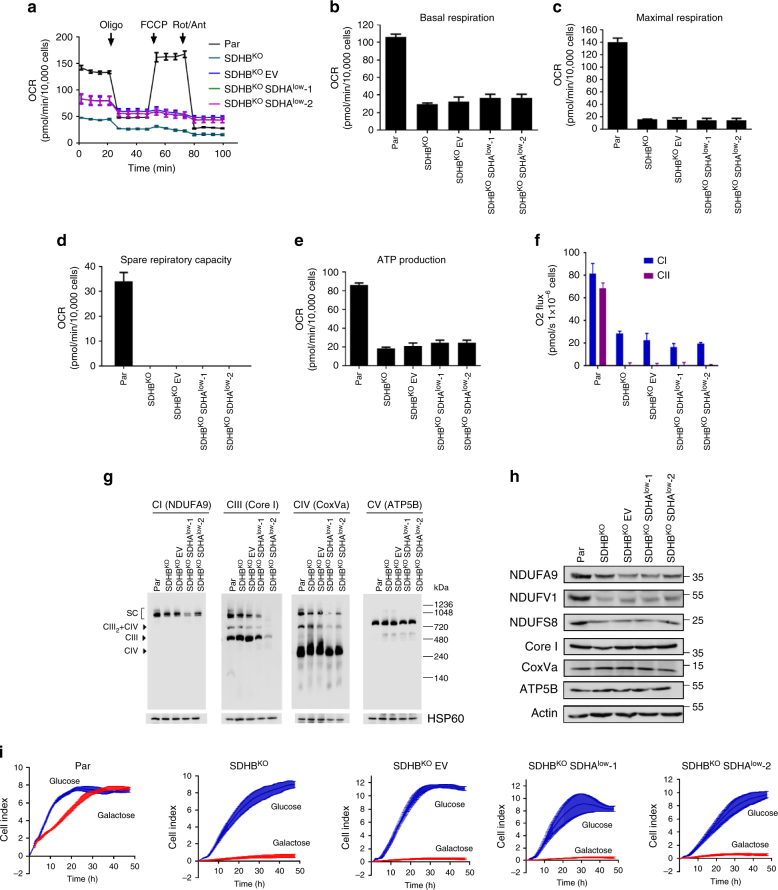
CII_low_ does not regulate mitochondial bioenergetics. **a** Oxygen consumption rate (OCR) in MDA231 sublines was followed during sequential additions of oligomycin, FCCP, and combination with rotenone and antimycin. **b**–**e** Basal respiration, maximal respiration, spare respiratory capacity, and ATP production. **f** CI- and CII-dependent respiration was evaluated in permeabilized cells using the Oxygraph. **g** Mitochondria isolated from MDA231 sublines were subjected to NBGE followed by WB analysis using antibodies against NDUFA9, NDUFV1, NDUFS8 (CI), Core I (CIII), and COXVa (CIV) and ATP5B (CV). **h** MDA231 sublines were analyzed for subunits of OXOPHOS complexes using WB after SDS-PAGE. **i** MDA231 sublines grown in media containing either glucose or galactose were evaluated for proliferation using the xCELLingence instrument. Data shown are mean values ± SD (*n* = 3); images and graphs in **i** are representative of three independent experiments

We asked whether altered assembly of SDHA affects mitochondrial bioenergetics as well as expression and assembly of other mitochondrial complexes. We found that basal respiration, maximum respiratory capacity, and ATP production were decreased both in SDHB^KO^EV and SDHB^KO^SDHA^low^ cells (Fig. 3a–e). Unlike parental cells, absence of spare respiratory capacity and low ATP production were found in SDHB^KO^EV and SDHB^KO^SDHA^low^ cells (Fig. 3d, e), further indicating a state of bioenergetic stress. We next assessed oxygen consumption in permeabilized MDA231 cells by high-resolution respirometry. This showed that alteration of the CII assembly status resulted not only in complete suppression of CII-dependent respiration, but also considerably altered CI-dependent respiration (Fig. 3f). Consistent with the above results, we found that assembly of SCs was reduced in SDHB^KO^ and SDHB^KO^SDHA^low^ cells (Fig. 3g). This is further supported by lower steady-state levels of CI subunits but not CIII, CIV, and CV subunits (Fig. 3h). However, there was no significant difference between SDHB^KO^ and SDHB^KO^SDHA^low^ cells for any of the assessed parameters. In contrast, re-expression of SDHB-FLAG (SDHB^rec^ cells) substantially recovered the parental phenotype with respect to routine and CII-dependent respiration (Supplementary Fig. S2f).

Cells with severe defects in OXPHOS rely on glycolysis for ATP production and do not grow in the presence of galactose^23^. We tested proliferation of MDA231 sublines in galactose- and glucose-containing media, and found that both SDHB^KO^EV and SDHB^KO^SDHA^low^ cells failed to proliferate in galactose-containing medium whereas parental cells proliferated efficiently (Fig. 3i). This is in agreement with previous reports on cells with defects in CI and CII assembly^23–26^. Again, re-expressed SDHB rescued proliferation in galactose-containing media (Supplementary Fig. 2g).

Similarly, analysis of mitochondrial morphology using transmission electron microscopy (TEM) revealed that both SDHB^KO^EV and SDHB^KO^SDHA^low^ cells featured mitochondria with altered structure (Supplementary Fig. 3a), consistent with a previous report^27^. These changes did not affect the steady-state level of several key proteins associated with mitochondrial maintenance and biogenesis (Supplementary Fig. 3b). Thus, while CII assembly status affected whole-cell bioenergetics and proliferation, CII_low_ had little additional effect on mitochondrial bioenergetics and biogenesis.

### Pyrimidine synthesis is attenuated in the presence of CII_low_

**Fig. 4:**
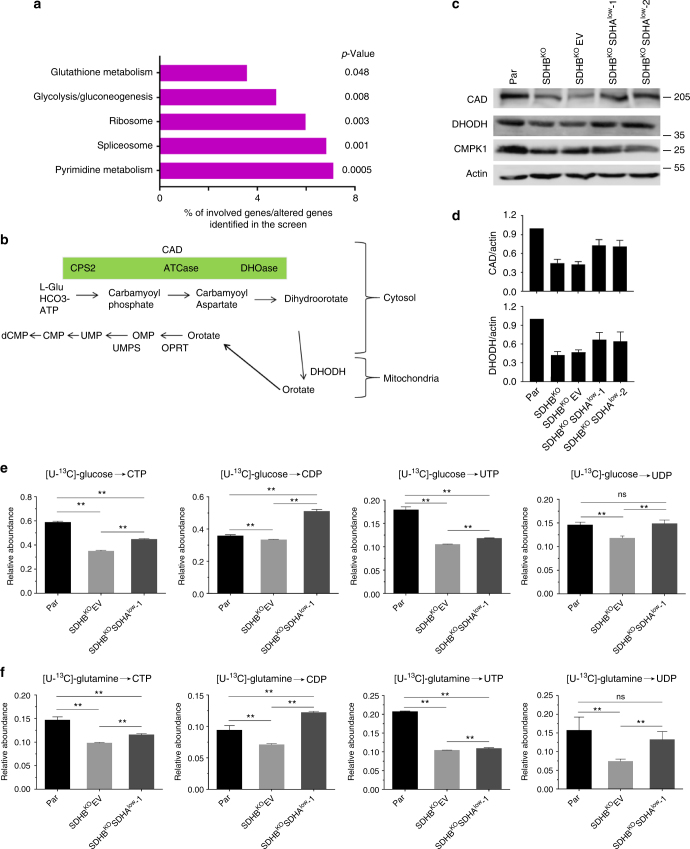
Pyrimidine biosynthesis is suppressed in the presence of CII_low_. **a** Label-free quantitative proteomic analysis of downregulated proteins in SDHB^KO^ MDAMB231 cells and the percentage of proteins identified per pathway relative to genes with significantly altered expression in SDHB^KO^ cells. **b** Scheme of the de novo pyrimidine nucleotide synthesis pathway. **c** SDS-PAGE following WB analysis of MDA231 sublines for proteins of the de novo pyrimidine pathway. **d** Densitometric evaluation of the level of the CAD and DHODH proteins by WB. Relative isotopomer amounts (M+1) of CTP, CDP, UTP, and UDP were assessed by LC-MS/MS using [U-^13^C] glucose (**e**) and [U-^13^C] glutamine (**f**) as tracers. Data shown are mean values ± SD (*n* ≥ 3); images are representative of at least three independent experiments. The symbol ** indicates differences with *p* < 0.05 and ‘ns’ indicates non-significant differences, as assessed by the two-tailed unpaired Student’s *t*-test

Having found no direct effect on bioenergetics, we reasoned that CII_low_ could modulate adaptation to altered bioenergetic conditions. Thus, we first performed quantitative proteomic analysis in MDA231 sublines using SWATH-MS^28^ that resulted in identification and quantification of 1699 individual proteins (Supplementary Data 2). The most downregulated pathway in SDHB^KO^EV cells compared to parental cells was de novo pyrimidine synthesis (Fig. 4a). Quantification of the SWATH-MS data revealed that this downregulation is reversed in SDHB^KO^SDHA^low^ cells (Supplementary Fig. 4), indicating that SDHB^KO^ cells may suppress anabolic processes to reduce the energy demand, and this is then relieved in SDHB^KO^SDHA^low^ cells. To further investigate this issue, we subjected parental, SDHB^KO^, and SDHB^KO^SDHA^low^ cells to RNAseq analysis (Supplementary Fig. 5a and b), which revealed significant clusters of genes either upregulated or downregulated in SDHB^KO^ cells with expression being reverted toward parental cell levels in SDHB^KO^SDHA^low^ cells (Groups 1 and 2 in Supplementary Fig. 5b–f). Gene set enrichment analysis revealed the presence of catabolism-related processes in these clusters, including heterocycle catabolic process (GO:0046700), aromatic compound catabolic process (GO:0019439), organic cyclic compound catabolic process (GO:1901361), and cellular macromolecule catabolic process (GO:0044265) that were all downregulated in SDHB^KO^SDHA^low^ cells compared to SDHB^KO^ cells (Supplementary Fig. 5e and f). However, the de novo pyrimidine synthesis pathway did not show significant enrichment, pointing to modest correlation of transcriptomic and proteomic data as previously reported^29–33^. Collectively, these data suggest that SDHB^KO^ cells may upregulate catabolic and salvage pathways to compensate for defects in pyrimidine biosynthesis.

We next focused our studies on pyrimidine biosynthesis. Initially, we evaluated representative proteins of this pathway using WB, and found that the trifunctional polypeptide CAD (carbamoyl-phosphate synthase 2, aspartate transcarbamylase, and dihydroorotase) and dihydroorotate dehydrogenase (DHODH) that catalyze the first four steps of de novo pyrimidine synthesis were attenuated in SDHB^KO^EV but recovered in SDHB^KO^SDHA^low^ cells (Fig. 4b–d). Re-expression of SDHB in SDHB^KO^ cells also restored CAD and DHODH (Supplementary Fig. 2h).

We then analyzed pyrimidine nucleotide synthesis directly using stable isotope labeling and LC-MS/MS. The data in Fig. 4e and f, and Supplementary Fig. 7, show that biosynthesis of CTP, CDP, UTP, and UDP from major nutrients glucose and glutamine was low in SDHB^KO^EV cells but reversed variably in SDHB^KO^SDHA^low^ cells. These results document that compared to the baseline situation (fully assembled CII), formation of CII_low_ is linked to depressed de novo pyrimidine synthesis, which is reversed with its depletion. The data suggest that under low-energy conditions, CII_low_ may activate cellular processes reducing ATP-consuming pathways such as DNA synthesis to maintain energy balance, which is deregulated in SDHB^KO^SDHA^low^ cells.

### CII_low_ plays a role in cell cycle progression

**Fig. 5:**
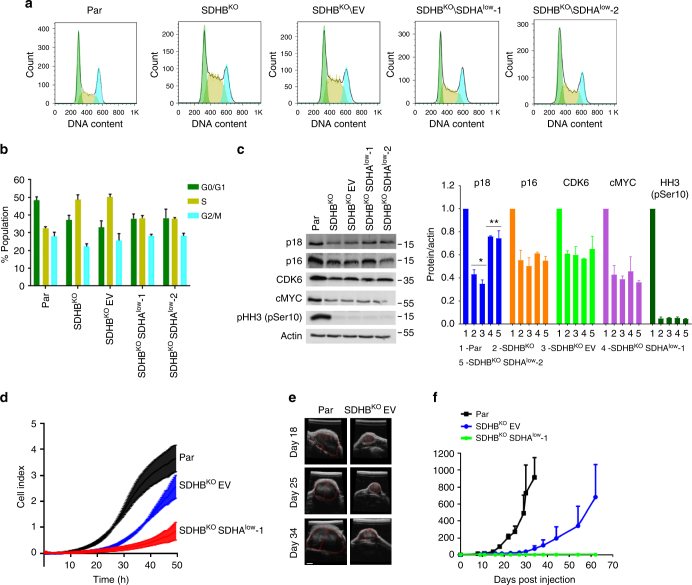
CII_low_ supports growth of SDHB-deficient tumors. **a** MDA231 sublines were evaluated for cell cycle distribution using flow cytometry and staining with propidium iodide. Green color indicates cells in G0/G1 phase, yellow cells in S phase, and blue color cells in G2/M phase. **b** Histograms show evaluation of data in panel (**a**) representing 10^4^ cells. **c** SDS-PAGE followed by WB analysis of proteins linked to the regulation of cell cycle progression and mitosis; images are representative of three independent experiments. **d** MDA231 sublines were assessed for migration capacity using the xCELLigence system. **e** MDA231 sublines were grafted in Balb-c nu/nu mice at 10^6^ cells per animal, and tumor progression was visualized and quantified by ultrasound imaging. The bar represents the size of 2 mm. **f** Representative images of tumors derived from parental and SDHB^KO^ EV cells. Data shown are mean values ± SD (*n* = 5 for **b** and **c**). The symbol * indicates significant differences compared to parental cells with *p* < 0.05 and ** significant differences compared to SDHB^KO^ cells with *p* < 0.05, as assessed by the two-tailed unpaired Student’s *t*-test

De novo pyrimidine synthesis is essential for maintaining the nucleotide pool for replication of DNA, thereby controlling cell cycle progression^34–37^. We thus analyzed cell cycle distribution in MDA231 sublines, which showed that SDHB^KO^ and SDHB^KO^EV cells were arrested in the S-phase. However, SDHB^KO^SDHA^low^ cells partially recovered from S-phase arrest with cell cycle distribution similar to that of parental cells (Fig. 5a and b). Cell cycle is regulated by cyclins and cyclin-dependent kinases (CDKs). WB confirmed a marked decrease in the level of CDK6 as well as p16, phosphorylated histone H3 (HH3), and cMYC in SDHB^KO^ and SDHB^KO^SDHA^low^ cells compared to parental cells (Fig. 5c). Interestingly, p18 was downregulated in SDHB^KO^ and SDHB^KO^EV cells and recovered in SDHB^KO^SDHA^low^ cells (Fig. 5c), similar to that seen for steady-state levels of CAD and DHODH and for nucleotide synthesis (Fig. 4). Further, we observed near parental levels of pHH3 in SDHB^rec^ cells (Supplementary Fig. 2h). This result suggests that alteration in SDHA assembly status affects de novo pyrimidine synthesis, and consequently cell cycle progression. Our data suggest that under low-energy conditions, the shift of CII to CII_low_ is associated with a switch to bioenergetically less-demanding processes.

### CII_low_ supports growth of SDHB-deficient tumors

Figure 2e reveals no difference in proliferation between SDHB^KO^EV and SDHB^KO^SDHA^low^ cells, suggesting that CII_low_ is not critical for proliferation under nutrient-rich conditions. However, imbalance of energy production and anabolic metabolism such as that linked to DNA synthesis could be critical for biomass production required for growth and survival under sub-optimal conditions^38^. To see whether CII_low_ is important for the tumorigenic potential of MDA231 sublines, migration of parental, SDHB^KO^EV, and SDHB^KO^SDHA^low^ cells was tested. Contrary to the cell proliferation assay (Fig. 2e), SDHB^KO^SDHA^low^ cells showed a lower rate of migration from serum-free media compared to both parental and SDHB^KO^ EV cells (Fig. 5d). To extend these results to a pathologically relevant situation, we tested the capacity of the sublines to form tumors. Parental, SDHB^KO^EV, and SDHB^KO^SDHA^low^ cells were grafted subcutaneously into Balb-c nu/nu mice, and tumor progression was analyzed by ultrasound imaging (USI). Compared to parental cells, SDHB^KO^EV cells formed tumors with a delay of about 15 days and at lower rate, while SDHB^KO^SDHA^low^ cells failed to form tumors 60 days post grafting (Fig. 5e and f). To understand whether the inability of SDHB^KO^SDHA^low^ cells to form tumors is linked to their higher vulnerability under nutrient-poor conditions, we evaluated the level of cell death in individual sublines grown in galactose media. Supplementary Fig. 3c, d shows that all sublines are mostly viable in glucose-containing media, while SDHB^KO^SDHA^low^ cells are more vulnerable to cell death than SDHB^KO^ cells in non-permissive, galactose-containing media. These data point to a link between CII_low_ and energy balance regulation to maintain cellular fitness under nutrient-poor conditions.

### CII_low_ modulates the metabolome

**Fig. 6:**
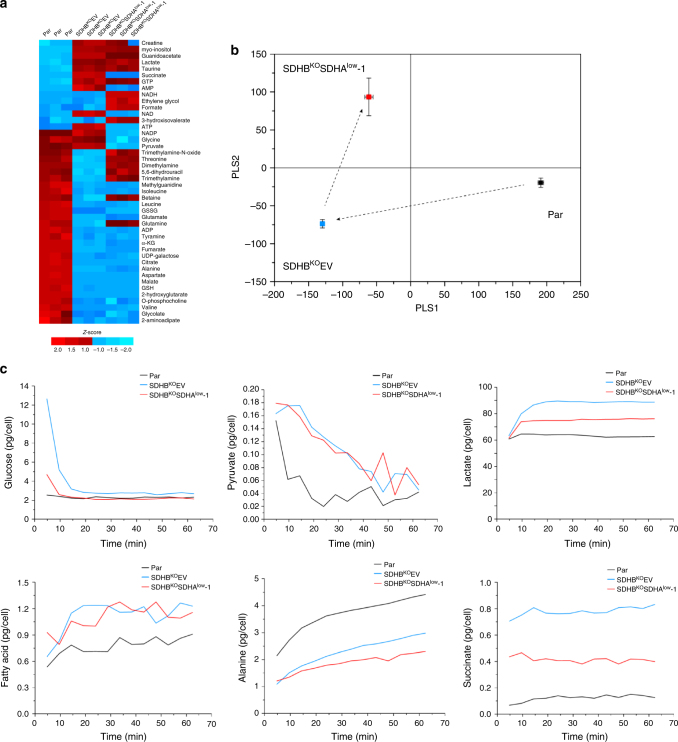
CII_low_ modulates metabolome of MDA231 sublines. **a** The heat map was constructed with the *Z*-score of peak intensities of each metabolite identified by both 1D NMR and LC-MS/MS from parental, SDHB^KO^EV, and SDHB^KO^SDHA^low^ cells. The *Z*-score was obtained by dividing the difference between actual peak intensities and mean value by the standard deviation. **b** Score plot from partial least squares discriminant analysis (PLS-DA) on MDA231 sublines with the entire 1D NMR spectral data. PLS1 axis accounts for the major metabolic discrimination (63.5%), and the PLS2 16.8%. The symbols represent the mean score values from the multivariate analysis and the whiskers represent one standard deviation. Transitions are shown by arrows. **c** Real-time flux comparison in live parental, SDHB^KO^EV, and SDHB^KO^SDHA^low^ cells. Time-dependent metabolic changes were obtained using 2D in-cell NMR metabolomics approach. The absolute quantification is based on the level of ^13^C carbon on a particular atom of metabolites detectable by NMR and was performed as described previously^39^. Although it is not possible to differentiate pre-existing natural abundance metabolites with those derived from ^13^C-glucose with real-time NMR, the natural abundance of ^13^C is 1% and pre-existing metabolites should not make significant contributions to the quantitation. Fatty acid represents the aggregate level of the ^13^C-labeled CH_2_ peak from free fatty acids at 1.36 and 32.0 ppm on the HSQC spectrum. Parental, black; SDHB^KO^EV, blue; SDHB^KO^SDHA^low^, red

In order to better understand metabolic consequences of alternative SDHA assembly, we examined the metabolic profiles of MDA231 sublines. Metabolomic data obtained by one-dimensional (1D) nuclear magnetic resonance (NMR) were combined with additional 16 metabolites, measured using targeted LC-MS/MS that are important in central energy metabolism but are not readily quantifiable by NMR (Supplementary Data 3). Results of this analysis, represented by the heat map in Fig. 6a, indicate an effect of CII_low_ on the differential metabolite profile in MDA231 sublines. The data show that the metabolic profile of SDHB^KO^EV cells has very little overlap with that of the parental cells, whereas SDHB^KO^SDHA^low^ cells have significantly more overlap. This is consistent with partial least square discriminant analysis (PLS-DA) of the metabolomic data showing the metabolic differences between sublines and the closer position of the SDHB^KO^SDHA^low^ cells to parental cells along the PLS1 axis (Fig. 6b). Taken together with the results in Fig. 1 which demonstrated CII_low_ formation during OXPHOS dysfunction, these data suggest that CII_low_ is linked to metabolic modulation when bioenergetics is compromised.

### Depletion of CII_low_ reverses accumulation of succinate

Given the role of SDHA in the TCA cycle, we expect that CII_low_ could affect succinate metabolism and TCA cycle activity. We therefore subjected parental, SDHB^KO^EV, and SDHB^KO^SDHA^low^ cells to two-dimensional (2D) in-cell NMR analysis, which allows monitoring of metabolites in live cells in real time^39^. The approach detects metabolite levels as a function of time, as opposed to steady-state levels at a single time-point as reported above and in most metabolomics studies. This facilitates estimation of actual activities of metabolic pathways leading to particular metabolites in live cells. Thus, cells were incubated with [U-^13^C]glucose, and production of ^13^C isotope-containing pyruvate, lactate, alanine, and the TCA cycle intermediate succinate was monitored. As expected, parental cells differed in all assessed metabolites from the two sublines. SDHB^KO^EV cells showed increased intracellular levels of lactate and alanine compared to SDHB^KO^SDHA^low^ cells, while fatty acid synthesis was not altered (Fig. 6c). Therefore, reduction of CII_low_ in SDHB^KO^SDHA^low^ cells seems to induce shuttling of pyruvate to pathways other than glycolysis without affecting fatty acid metabolism. Importantly, SDHB^KO^EV cells had a higher level of succinate than parental cells, in agreement with previous reports for other SDHB^KO^ models^40–44^. However, SDHB^KO^SDHA^low^ cells showed reduced succinate levels compared to SDHB^KO^EV cells (Fig. 6c), suggesting a CII_low_-dependent change in succinate metabolism, which may be due to activation of an alternative metabolic route of succinate utilization.

### CII_low_ negatively regulates anabolic activity of TCA cycle

**Fig. 7:**
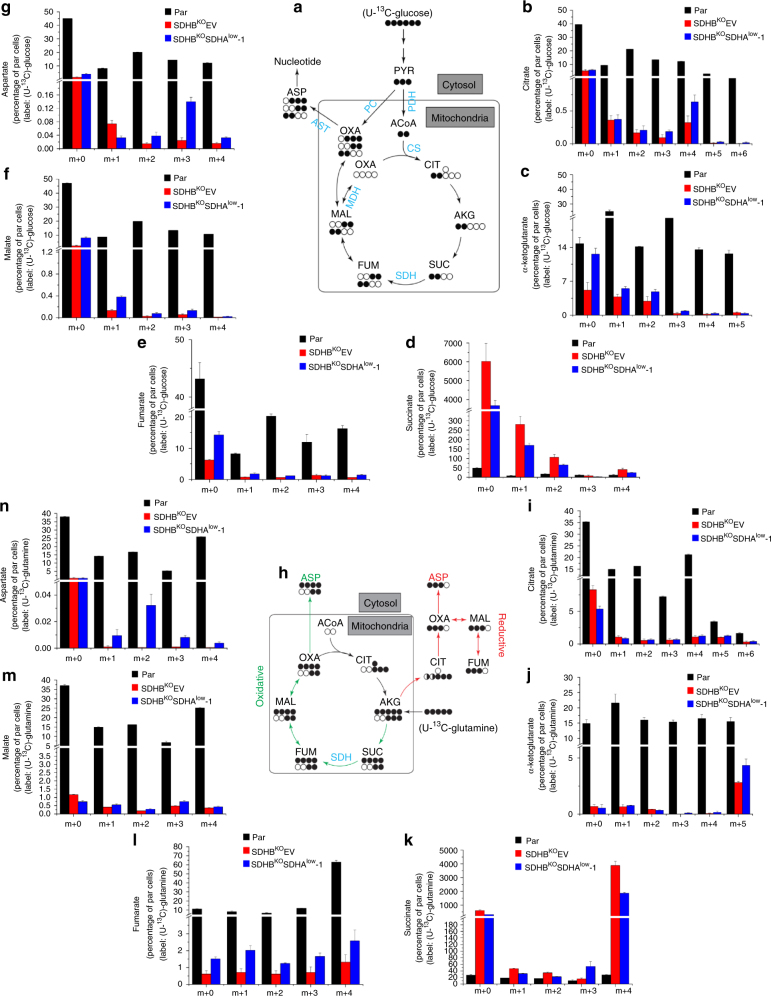
CII_low_ restricts TCA-linked anabolism. **a** and **h**, Predicted labeling patterns of indicated metabolites from U-^13^C_6_-glucose (**a**) and from U-^13^C-glutamine (**h**) (filled circles ^13^C; open circles ^12^C). The oxidative (green) and reductive (red) carboxylation pathways are indicated (**h**). Isotopologue distribution of intracellular citrate (**b**, **i**), a-ketoglutarate (**c**, **j**), succinate (**d**, **k**), fumarate (**e**, **l**), malate (**f**, **m**), and aspartate (**g**, **n**) after incubation for 24 h with 5 mM unlabeled glucose and 20 mM U-^13^C-glucose or 4 mM U-^13^C-glutamine, respectively. The data were obtained using LC-MS/MS analysis. The *Y* values are presented as the percent values of the sum of all the isotopologues per each metabolite in parental cells; ‘m + …’ indicates the relevant isotopomer. Data are presented as mean ± SD, *n* = 3. PYR pyruvate, ACoA acetyl-CoA, CIT citrate, AKG α-ketoglutarate, SUC succinate, FUM fumarate, MAL malate, OXA oxaloacetate, ASP aspartate, PDH pyruvate dehydrogenase, PCX pyruvate carboxylase, CS citrate synthase, SDH succinate dehydrogenase, MDH malate dehydrogenase, AST aspartate transaminase

To obtain further insight into succinate metabolism as well as metabolism of other TCA cycle intermediates, we monitored the fate of [U-^13^C] glucose and [U-^13^C] glutamine in MDA231 sublines using LC-MS. The first cycle of TCA metabolism of [U-^13^C] glucose via acetyl-CoA will generate four-carbon metabolites with two ^13^C nuclei (m+2 isotopomer) through oxidative decarboxylation (Fig. 7a–f). Alternatively, an m+3 isotopomer can be formed if glucose enters the TCA cycle via the pyruvate carboxylase (PC) pathway (Fig. 7a). In agreement with the above NMR data (Fig. 6c), we observed increased m+2 isotopomers of succinate in SDHB^KO^EV cells, and this was reduced in SDHB^KO^SDHA^low^ cells (Fig. 7d). Concurrently, the levels of m+2 isotopomers of aspartate, malate, and fumarate increased in SDHB^KO^SDHA^low^ cells compared to SDHB^KO^EV cells (cf. Fig. 7e–g), suggesting more efficient consumption of succinate in cells with lower levels of CII_low_. The increase in m+3 isotopomer of aspartate and malate in SDHB^KO^SDHA^low^ cells, but not that of fumarate (Fig. 7e–g), suggests that the PC pathway may also contribute to the four-carbon metabolites in these cells.

[U-^13^C] glutamine feeds carbons into the TCA cycle via α-ketoglutarate. A subsequent oxidative decarboxylation in the TCA cycle generates the m+4 isotopomer of succinate that can lead to m+4 aspartate (Fig. 7h, j–n). Succinate and aspartate m+2 isotopomers can also be formed through the TCA cycle if the m+4 succinate condenses with acetyl-CoA, followed by oxidative decarboxylation in subsequent TCA cycles (Fig. 7h, green). [U-^13^C] glutamine can also contribute to formation of the m+3 isotopomer of aspartate via reductive carboxylation (Fig. 7h, red). In parental cell lines, we observed simultaneous oxidative decarboxylation and reductive carboxylation of glutamine as shown by high levels of incorporation of [U-^13^C] glutamine into m+2, m+3, and m+4 aspartate isotopomers (Fig. 7n), consistent with reports on other cancer cells^45–48^. Significant formation of m+5 citrate isotopomer also suggests reductive carboxylation in parental cells (Fig. 7i). We observed very high absolute incorporation of [U-^13^C] glutamine into m+4 succinate in SDHB^KO^EV and a significant decrease to about half in SDHB^KO^SDHA^low^ cells (Fig. 7k). These were accompanied by higher incorporation of [U-^13^C] glutamine into m+4 aspartate and fumarate in SDHB^KO^SDHA^low^ cells than SDHB^KO^EV cells (Fig. 7l and n). Similar patterns were observed for the m+2 succinate isotopomer (Fig. 7k), although the absolute incorporation was much lower, probably due to a second round of the TCA cycle. These glutamine incorporation data clearly indicate that glutamine-derived succinate accumulates in SDHB^KO^EV cells, whereas it is converted to fumarate and ultimately to aspartate more efficiently in SDHB^KO^SDHA^low^ cells.

Overall, both glucose and glutamine isotope incorporation experiments indicate a block in succinate metabolism in SDHB^KO^EV cells that is at least partially lifted when the CII_low_ is depleted on the SDHB^KO^ background (Supplementary Fig. 5a and c). This phenomenon is also consistent with the reverse trend of fumarate, whose level increased in SDHB^KO^SDHA^low^ cells (Supplementary Fig. 5b and d). This re-flow of succinate is suggested to induce redistribution of carbon atoms of glucose and glutamine to four-carbon metabolites leading to the recovery of synthesis of aspartate, a precursor for pyrimidine synthesis.

Previous studies showed that CII dysfunction caused by SDHB–SDHD mutations/deficiency results in accumulation of succinate^2,40,42–44,49,50^. Here we demonstrate that SDHA maintains high levels of succinate following CII dysfunction. Our data also implicate a switch of carbon metabolism in a CII_low_-dependent manner, mainly from succinate accumulation to anabolic reactions, including synthesis of aspartate to produce pyrimidines. Further investigations are needed to elucidate the enzymatic activity of CII_low_, especially in the context of succinate accumulation due to reductive carboxylation of glutamine (Fig. 7h).

### CII_low_ is a feature of SDHB-deficient paraganglioma

**Fig. 8:**
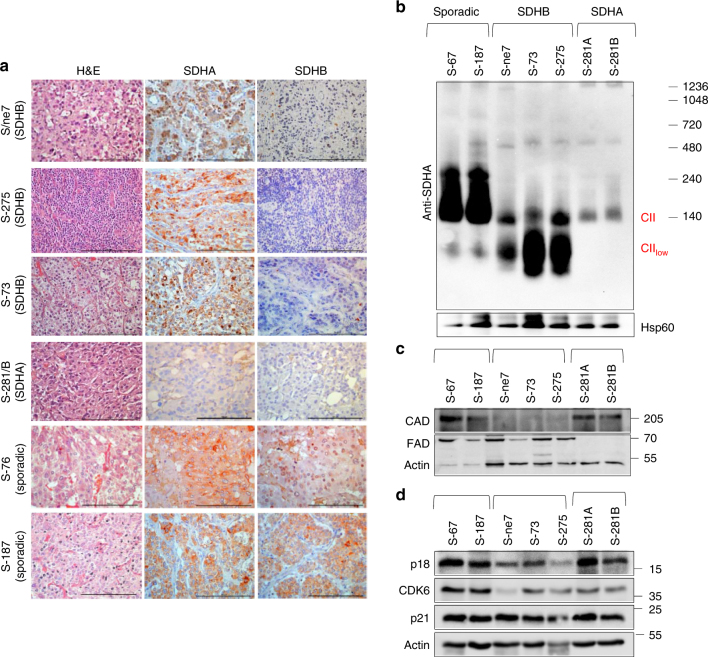
CII assembly and de novo pyrimidine pathway in paragangliomas. **a** Histological examination of SDHA and SDHB expression from tumor tissue of paraganglioma patients with *SDHA* or *SDHB* mutations or with sporadic paragangliomas. Tissues were processed for H&E staining and immunohistochemistry using SDHA and SDHB IgGs. Representative images are collected using a 40× objective. Scale bars represent 100 μm. **b**–**d** Paraganglioma tissues, as shown, were assessed by WB following protein separation by NBGE for CII assembly using anti-SDHA IgG (**b**), and by SDS-PAGE for CAD and FAD (**c**) and for markers of cell cycle (**d**). Images are representative of three independent experiments

To assess the clinical relevance of our findings, we inspected tumor tissue from paraganglioma patients with *SDHA* and *SDHB* mutations, as well as sporadic paraganglioma patients. We assessed tumors from six patients for the presence of SDHA and SDHB using immunohistochemistry. Fig. 8a shows that samples from patients with mutated *SDHB* exhibit high levels of SDHA, while tumor tissue derived from a patient with *SDHA* mutation showed very low levels of both SDHA and SDHB proteins. Next, tumors of these patients were evaluated for CII assembly and for the level of de novo pyrimidine synthesis. The rate-limiting trifunctional CAD protein as well as cell cycle regulatory proteins were assessed. NBGE showed high levels of CII_low_ and low levels of fully assembled CII in tumors with *SDHB* mutations, while tumors with *SDHA* mutations showed low levels of fully assembled CII and the absence of CII_low_ (Fig. 8b). Further, *SDHA*-mutated paragangliomas lacked FAD, a cofactor of CII associated with the catalytic subunit SDHA, while it was detected in sporadic paragangliomas or those with *SDHB* mutations. Paragangliomas with high levels of Cll_low_ were found to contain low levels of CAD and p18 (Fig. 8c and d). These data are in agreement with results found for sublines of MDA231 cells with different CII assembly status (Fig. 2d; Fig. 4c; Fig. 5c). The data imply that CII_low_ may vary under different (patho)physiological conditions, giving our findings clinical relevance.

## Discussion

In the present study, we show that alternative assembly of CII fine-tunes cellular metabolic homeostasis to compensate for chronic bioenergetic stress. SDHA is known to exist in a complex with other subunits of CII, with an overall molar mass of 124 kDa, migrating on native gels at ~140 kDa^22,51^. We found an assembly form of SDHA migrating at ~100 kDa (designated here as CII_low_; Figs. 1 and 2), lacking SDHB and SDHC. This is consistent with previous studies using an experimental model of yeast, mammalian cell culture, and human pathological conditions showing that SDHA is stable in the absence of SDHB^40–44,52^, although its biological function has never been investigated. In addition, CII_low_ is more prevalent when mtDNA is depleted or its expression is compromised (Fig. 1). This suggests biological relevance of CII_low_ with mtDNA mutations and under conditions that limit mitochondrial energy production. We propose that CII/CII_low_ has a role in mitochondria similar to that of pyruvate kinase 2 (PKM2), which changes its assembly from a highly active tetrameric to low-active dimeric form in response to different cellular signaling pathways. A switch from the tetrameric to dimeric form of PKM2 has profound consequences for cellular metabolism as well as proliferation in the context of diabetic nephropathy^53^ and tumorigenic capacity of cancer cells^54–56^.

The molecular weight of SDHA is about 70 kDa and thus there are likely to be other SDHA-interacting proteins in CII_low_ of approximately 100 kDa. To identify SDHA-interacting proteins, we used immunoprecipitation followed by MS analysis. This analysis, supported by WB, identified the assembly factors SDHAF2 and SDHAF4 as proteins that together with SDHA constitute CII_low_ accounting for its ~100 kDa mass (Supplementary Fig. 2). We have recently reported that SDHAF2 has redundant function in relation to CII activity in MDA231 cells^57^. While we do not know the function of SDHAF2 and SDHAF4 in CII_low_, it is possible that these factors form a functional association with SDHA within CII_low_ and in the absence of the other three subunits (SDHB–D). The role of SDHAF2 and SDHAF4 in CII_low_ is the subject of current investigation.

To study the cellular function of CII_low_, we established stable cellular models with three different assembly forms of SDHA (Fig. 2) and initially analyzed bioenergetics difference of these sublines. We found that SDHB^KO^ cells with prominent presence of CII_low_ switched to low basal respiration with no spare respiratory capacity, indicating an energy stress situation. SDHB^KO^SDHA^low^ cells with depleted CII_low_ showed a bioenergetic pattern similar to that of SDHB^KO^ cells (Fig. 3), indicating that CII_low_ plays only a minimal role in mitochondrial bioenergetics.

Label-free proteomic and pathway analysis allowed for identification of differences in the de novo pyrimidine pathway in MDA231 sublines. We found that CII_low_ abundance is inversely correlated with de novo pyrimidine pathway activity (Fig. 4), as exemplified by the steady-state level of CAD, a trifunctional and rate-limiting polypeptide in the pathway. The level of the CAD protein was reduced by approximately 60% in SDHB^KO^ cells resulting in cell cycle arrest in the S-phase, and its re-expression in SDHB^KO^SDHA^low^ cells with depleted CII_low_ normalized cell cycle progression (Fig. 5). Recently, the de novo pyrimidine synthesis pathway has been shown to be essential in metabolic rewiring that modulates chemotherapy resistance of triple-negative breast cancer^58^ and other neoplastic pathologies^59,60^, supporting the notion that de novo pyrimidine synthesis improves plasticity of cancer cells at the expense of increased energy consumption. Further investigations will be needed to provide unequivocal evidence for a functional linkage between CII_low_ and pyrimidine biosynthesis. However, our data suggest that CII_low_ exerts its protective function not by modulating mitochondrial bioenergetics (i.e., OXPHOS), but through other mitochondria-related mechanisms, which are not yet fully defined but seem to involve energy conservation strategies such as a reduction of the de novo pyrimidine synthesis pathway. Therefore, we propose that restriction of this pathway and possibly of other energy-demanding processes by Cll_low_ may provide a selective advantage under suboptimal nutrient conditions when bioenergetics is compromised, exemplified by increased cell death in galactose media and a the failure to form tumors in a mouse xenograft model (Fig. 5) when SDHB-deficient cells lose CII_low_. In line with this, CII_low_ is less abundant in optimal physiological conditions and becomes the dominant SDHA form of CII during sub-optimal energy production (Fig. 1).

Our data suggest that deficiency of CII assembly and the shift of SDHA to CII_low_ modulates metabolism in response to compromised mitochondrial bioenergetics. It introduces checkpoints of cellular functions with high demands for energy, for example reducing formation of aspartate that is essential for pyrimidine nucleotide synthesis. This indicates that CII_low_ might be a sensor of bioenergetic stress, providing a feedback from OXPHOS to central carbon metabolism. Similarly, a number of proteins capable of sensing mitochondrial energy production or metabolite levels that reflect energy status have been identified. AMP-activated protein kinase (AMPK) has been shown to sense the ratio of ATP to ADP and AMP level in order to initiate an energy compensating stress response^61,62^. Previous studies have also demonstrated that certain sirtuins are sensors of organelle energy production in relation to the NADH/NAD^+^ ratio^63^. Recently the ubiqunol/ubiqunone ratio has been reported to sense mitochondrial energy production linked to the respiratory status^64^. Possible links between CII assembly forms and other sensors of mitochondrial bioenergetic dysfunction need to be further investigated. Merging these various individual components into a highly integrated regulatory system presents the next challenge.

Data shown here reveal that patients with *SDHB* and *SDHA* mutations have different CII assembly status. We found that CII_low_ is the remaining unit of CII present in patients with *SDHB* mutations, but is absent in patients with SDHA and sporadic mutations. In agreement with our cellular model of MDA231 sublines, patients that show high levels of CII_low_ also have reduced levels of the de novo pyrimidine synthesis regulatory enzyme CAD and the cell cycle modulator protein p18 (Fig. 8). Clinical data also indicate that SDHB mutation-associated paragangliomas are less proliferative but are associated with higher invasiveness and metastasis^42,65–67^. This may be one of the reasons why SDHA-deficient tumors are relatively rare, possibly due to an imbalance in energy and metabolic homeostasis. Thus, our data provide more detailed insights into established clinic-pathological features for incurable *SDHx*-related paragangliomas and may potentially serve as a diagnostic marker.

In summary, we found that following mtDNA depletion or SDHB deficiency, an alternative CII with different activity is formed. We suggest that the existence of CII_low_ in patients with *SDHB*-mutated paragangliomas may contribute to a cellular mechanism resulting in severe pathological outcomes, including enhanced migration and invasiveness. Hence, SDHA epitomizes a moonlighting protein, being an enzyme that is a baseline tumor suppressor but becomes a promoter of tumor growth in the context of bioenergetic deficiency.

## Methods

### Cells and transfection

MDA231 and MCF7 cells were obtained from the ATCC and cultured in DMEM (Life Technology) supplemented with 10% FCS and antibiotics plus antimycotics (Gibco). To generate SDHB^KO^ cell lines, MDA231 cells were transfected with the TALEN construct obtained from Genecopoeia. Transfections were performed using Lipofectamine 2000 (Invitrogen) according to the manufacturer’s instructions; transfectants were selected with hygromycin. Two shRNAs targeting the human *SDHA* gene and empty vector (EV) were purchased from OriGene. SDHB^KO^ cells were transfected with the shRNA plasmids. After 36 h, cells were treated for 5 days with 0.8 µg/ml puromycin. Preparation of mtDNA-depleted cells (4T1ρ^0^ and MCF7ρ^0^) was accomplished by their long-term incubation with 50 μM EtBr^13,57^. Human SDHB was re-expressed in SDHB^KO^ cells from the pLYS5-SDHB-Flag plasmid^68^ (Addgene # 50055, a kind gift of Vamsi Mootha) using lentiviral transduction. Lentivirus particles were produced in Hek293T cells using second generation psPAX and pMD.2G plasmids and lipofection (Lipofectamine 3000, Invitrogen). Virus-containing media were collected after 48 h, centrifuged at 3000×*g* for 15 min and stored at −80 °C^69^.

### Proliferation, migration, and cell death assays

Parental, SDHB^KO^EV, and SDHB^KO^SDHA^low^ cells were plated in a 24-well dish in DMEM. At indicated times, cell proliferation was assessed by crystal violet assay using a standard protocol. xCELLigence Real-Time Cellular Analysis system was used to evaluate cell proliferation in glucose- or galactose-containing medium and using dialyzed FBS (Gibco). For this, cells were seeded in 16-well E-plates (4 replications per cell type) as recommended by the manufacturer. For migration assays, cells were transferred to the CIM-16 plates and allowed to migrate toward FBS placed in the other compartment. Cell proliferation was monitored in real time using the xCELLigence instrument for 50 h, and the cell index was recorded every 5 min. Cell death was assessed using the standard annexin V/propidium iodide (PI) method.

### siRNA transfections

Mouse Silencer-select pre-designed SDHA-, SDHB-, and SDHC-targeting siRNAs and non-silencing controls were obtained from Life Technologies. The sequences are as follows: SDHA target sequence-1 (s84146): GGA ACA CUC CAA AAA CAG Att; SDHA target sequence-2 (s211850): CCA GUU AUU UUG UGG AAU Att; SDHB target sequence-1 (s205969): GCU UUA AUC AAG AUC AAG Att; SDHB target sequence-2 (s205970): CCC UCU UCC ACA UAU GUA Utt; SDHC target sequence-1 (s82476): GAU CUA CUC GGC UAA GUU Utt; SDHC target sequence-2 (s205071): GAA CAC GAG UUC AAA CCG Utt. 4T1 cells were transiently transfected with 20 nM siRNA using Lipofectamine RNAiMAX transfection reagent (Invitrogen) according to the manufacturer’s instructions and harvested for analysis 72 h after transfection.

### Cell cycle analysis

Cells were harvested with trypsin, rinsed with PBS, fixed by drop-wise addition into 70% (v/v) ethanol and kept at −20 °C for at least 4 h. They were then centrifuged, rinsed in PBS, re-suspended in PBS containing 80 μg/ml RNase and 30 μg/ml of propidium iodide plus 100 μg/ml RNase A. After incubating for 30 min at room temperature, DNA content was assessed using the FACS Calibur flow cytometer (Becton Dickinson). Cell cycle distribution was estimated using the FlowJo software and the Watson distribution model.

### Western blotting

Cells were lysed in 20 mM Tris (pH 8), 200 mM NaCl, 1 mM EDTA, 0.5% (v/v) NP-40, and 10% glycerol supplemented with protease inhibitors (Roche). After addition of the Laemmli solution to the samples, the proteins were separated by SDS-PAGE (8–15% gel) and then transferred to polyvinylidene difluoride (PVDF) membranes. After blocking with 5% non-fat milk, the blots were incubated with the primary antibodies overnight at 4 °C. The membranes were then treated with horseradish peroxidase-conjugated secondary antibodies for 2 h at room temperature, followed by visualization (SuperSignal West Pico Chemiluminescent Substrate, Pierce). The following antibodies were used in the study: SDHA (Abcam, ab14715; 1:2000); SDHB (Abcam, ab14714; 1:1000); SDHC (Abcam, ab155999; 1:1000); HSP60 (Abcam, ab137706; 1:2000; or Cell Signaling, 12165; 1:2000); CoxVa (Abcam, ab110262, 1:2000); NDUFA9 (Life Technologies/Thermo Fisher, 459100; 1:2000); Core I/UQCRC1 (Life Technologies/Thermo Fisher, 459140; 1:2000); NDUFV1 (Abcam, ab55535; 1:1000); NDUFS8 (Abcam, ab170936; 1:1000); SDHAF2 (Cell Signaling, 45849; 1:1000); SDHAF4 (Thermo Fisher, PA5-73014; 1:1000; or Sigma, HPA031824; 1:500); FAD (MyBioSource, MBS2015613; 1:500); HRP-conjugated tubulin (Thermo Scientific, MA5-16308-HRP; 1:2000); HRP-conjugated actin (Abcam, ab49900; 1:5000); actin (Abcam, ab14715 or 3700, or Cell Signaling, 4970 or 3700; all 1:3000); DHODH (Proteinech, 14877-1-AP; 1:500); CAD (Cell Signaling, 93925; 1:1000); phospho-Histone H3 (Ser10) (Cell Signaling, 3642; 1:1000); GAPDH (Cell Signaling, 8337; 1:3000). Uncropped western blots are in Supplementary Fig. 8.

### Isolation of mitochondria and NBGE

Mitochondria were isolated using Dounce or Balch homogenizers, followed by standard differential centrifugation^13,50,70^. Experimental procedure and antibodies for NBGE were used as described previously^57,71^. In brief, digitonin-solubilised mitochondria were separated on NativePAGE Novex Bis-Tris 3–12% gradient gels. After electrophoresis, the gels were incubated in transfer buffer containing 0.1% SDS for 10 min and proteins were transferred to PVDF membranes probed with specific antibodies (all diluted 1:500, except for VDAC1 and HSP60 diluted 1:1000) against complex I (NDUFA9, ab14713, Abcam; or NDUFB8, ab110242, Abcam), CII (SDHA, 14715, Abcam), CIII (Core2, ab14745, Abcam), CIV (COXVa, ab110262, Abcam) and CV (ATP5A, ab14748, Abcam; or ATP5B, HPA001520, Sigma Aldrich), and VDAC1 (ab15895, Abcam) or HSP60 (12165S, Cell Signaling) as the loading control.

### Evaluation of oxygen consumption rate

Cells were seeded into an XFp assay microplate (Agilent) 1 day prior to evaluation. On the day of the assay, the growth medium was replaced with the XF assay medium (Agilent) supplemented with glucose and glutamine. Oxygen consumption rate (OCR) measurements were made using the Seahorse Analyzer (Agilent) with sequential addition of oligomycin, FCCP, rotenone and antimycin A (using the Mitostress kit from Agilent) according to the manufacturer’s protocol. OCR measurements were adjusted based on the cell number counted at the end of the experiment.

### Respiration assays

Respiration was evaluated in digitonin-permeabilized MDA231 sublines and assessed as described previously.^72,73^ In brief, the cells were trypsinized, washed with PBS, re-suspended at 1 × 10^6^ cells per ml of the Mir05 medium (0.5 mM EGTA, 3 mM MgCl_2_, 60 mM K-lactobionate, 20 mM taurine, 10 mM KH_2_PO_4_, 110 mM sucrose, 1 g/l essentially fatty acid-free bovine serum albumin, 20 mM Hepes, pH 7.1 at 30 °C) and transferred to the chamber of the Oxygraph-2k instrument (Oroboros). Respiration measurements were performed at 37 °C. Cells were permeabilized with 5 µg digitonin per 10^6^ cells, followed by sequential additions of substrate and inhibitors. CI respiration was assessed in the presence of glutamate/malate and ADP, while CII respiration in the presence of succinate, ADP, and rotenone.

### Xenograft experiments

All procedures with animals were performed according to the Institutional guidelines and ethical authorization by the Griffith University Animal Ethics Committee. MDA231 sublines were injected into the mammary fat pad of Balb-c nu/nu mice at 10^6^ cells per animal. Tumor volume was quantified using the Vevo3100 USI system using a 30-μm resolution scan-head^70,74^.

### SWATH proteomic analysis

For proteomic analysis, cell pellets were lysed using 200 µl of sodium deoxycholate buffer (0.1% in 0.1 M triethyl ammonium bicarbonate). Following reduction with 5 mM dithiothretol and alkylation with 10 mM iodoacetamide, 100 μg of protein was digested with sequencing grade trypsin (Promega) at 37 °C for 16 h. The sample was acidified using formic acid and centrifuged for 10 min to remove the precipitated sodium deoxycholate salt. Tryptic peptides were recovered and fractionated using High pH Reversed-Phase Peptide Fractionation Kit (Pierce) according to manufacturer’s instructions, with the exception that only six fractions were collected. To establish a reference spectral library for SWATH analysis, the fractionated sample was run by nanoLC-MS/MS using a 100 mm × 150 µm C18 column coupled to an Eksigent Ultra system over 90 min as described^75^ using Information-Dependent Acquisition (IDA) on a 5600+ Triple TOF mass spectrometer (Sciex, Framingham, MA) using the Top 10 most intense multiply charged ions. MS/MS was conducted for 50 ms over the 100–1500 *m/z* range. Peptides were identified using ProteinPilot (v4.2) (Sciex) to search the UniProt Human protein database (20,198 entries, downloaded June 2015) and false-discovery controlled by searching a reversed-decoy Human database of identical size, selecting >99% confidence for protein identification. The Paragon group file was imported into PeakView software 2.1 using the SWATH MicroApp 2.0 to generate a sample specific spectral library.

For SWATH data acquisition of individual samples, we used the same MS setup but adjusted the method to use 60 variable *m/z* windows (400–1250 *m/z*) for precursor ion selection. The MS/MS spectra were accumulated for 60 ms in the *m/z* 350–1500 *m/z* range.

To extract SWATH peak areas with PeakView software 2.1, we carried out retention time calibration with endogenous peptides and data processing using following settings; 100 maximal peptides per protein, maximal 6 transitions per peptide, peptide confidence threshold of 99%, transition false discovery rate <1%, 10 min extraction window, and fragment extraction tolerance of 75 ppm, exclusion of shared peptides. The protein peak areas were normalized to the total peak area and log-transformed peak areas and subjected to Student’s *t*-test to compare relative protein peak area between samples. Proteins were considered to be differentially expressed with *p* < 0.05 and protein fold change was ±1.5 fold. DAVID^76^ was used for functional enrichment analysis. The Benjamini method (adjusted *p* value) was used to control the family-wide false discovery rate for enrichment analysis.

### Identification of SDHA-interacting proteins

Mitochondria isolated from SDHB^KO^ cell stably expressing SDHA-FLAG and empty vector were lysed using membrane solubilization buffer (1% *n*-dodecyl *β*-D-maltoside, 20 mM Tris-HCl, pH 7.4, 0.1 mM EDTA, 100 mM NaCl, 10% glycerol) supplemented with protease inhibitors (Roche). Protein concentrations were determined using the BCA protein assay kit. Equal amounts of protein were incubated with 20 μl anti-FLAG M2 agarose beads (Sigma) overnight at 4 °C. Beads were washed three times with the membrane solubilization buffer. The proteins bound to the beads were then eluted with 2× SDS lysis buffer and separated by 15% SDS-PAGE followed by silver staining (Mass spectrometry compatible, Life Technologies). Protein bands from control and treatment lanes were subjected to tryptic digest and analyzed by mass spectrometry.

### Sample preparation for NMR spectroscopy and LC-MS

Metabolites were extracted from parental, SDHB^KO^EV, and SDHB^KO^SDHA^low^ cells (3 × 10^6^ cells) with 400 µl of mixture composed of methanol, acetonitrile, and distilled water (5:3:2). The samples were centrifuged at 15,000×*g* for 20 min at 4 °C. The supernatant was collected, divided into two portions at the ratio of 1:5 for LC-MS and NMR analysis, and dried with a vacuum centrifuge (Vision). The pellets for LC-MS were dissolved with 30 µl mixture of HPLC-grade acetonitrile and water (1:1), and those for NMR with 500 µl buffer composed of 2 mM Na_2_HPO_4_ and 5 mM NaH_2_PO_4_ in D_2_O with 0.025% TSP (trimethylsilylpropionic acid sodium salt–D_4_) as an internal standard. For the isotopologue distribution analysis, these cells were cultured in glucose and glutamine-free DMEM media (Gibco) supplemented with 10% dialyzed FBS (Welgene, Daegu, Korea), 10% D_2_O, 5 mM unlabeled glucose, and 20 mM U-^13^C_6_-labeled glucose (Cambridge Isotope Laboratories) or 4 mM U-^13^C_5_-labeled glutamine (Cambridge Isotope Laboratories) for 24 h before metabolites extraction, respectively.

### Measurement and analysis for NMR spectroscopy and LC-MS

Untargeted metabolomic profiling was performed using NMR, and targeted profiling by LC-MS multiple reaction monitoring for metabolites that are not readily discernible by NMR. Metabolites detected by both methods, such as succinate and glutamate, exhibited consistent results. 1D NMR spectra were obtained using a 500-MHz Bruker Avance spectrometer equipped with a cryogenic triple resonance probe (KBSI). For the pulse program, ‘noesygppr1d’ was used with 64 scans, and the final spectra were constructed to 16K points. The metabolites were identified with Chenomx spectral database (Edmonton, Alberta, Canada) and comparison with standard compounds^39^. LC-MS data were obtained with an LTQ XL high performance linear ion trap mass spectrometer (Thermo Fisher Scientific) equipped with an electrospray ionization source. The operating conditions of the mass spectrometer were as follows: 5 kV of ion spray voltage, heated capillary temperature of 275 °C, and sheath gas (nitrogen), auxiliary gas (nitrogen), and sweep gas (nitrogen) pressures of 35, 10, and 2 (arbitrary units), respectively. Full scanning analyses were performed in the range of *m/z* 85–1000, and a 35-V normalized collision energy was used for MS/MS. Mobile phases were 10 mM ammonium carbonate (pH 9.1) in distilled water (A) and acetonitrile (B), and the flowrate was 0.15 ml/min. The gradient scheme was as follows: 80% B at 0 min, 35% B at 10 min, 5% B at 12 min, 5% B at 25 min, 80% B at 25.1 min, and 80% B at 35 min. The Zic-pHilic Polymeric Beads Peek Column (150 × 2.1 mm, 5 μm; Merck) was used at 35 °C, and the autosampler temperature was set at 4 °C. The peak areas of parental ion and its isotopologues were measured using Xcalibur (Thermo Fisher Scientific). The metabolites were identified by m/z values and MS/MS fragmentation patterns from Human Metabolome Database (HMDB) and METLIN databases and also by comparison with standard compounds^77^. For multivariate analysis, the NMR data were Fourier-transformed, phase-corrected, and baseline-corrected manually using MestReNova (Mestrelab Research, Santiago de Compostela, Spain). The signal intensities were normalized against the intensity of the 0.025% TSP signal at 0 ppm and total area values. The processed NMR data were saved into a text file and binned at a 0.004-ppm interval. The water region (4.66–5.0) was excluded from the spectra. The binning and normalization were performed using a Perl software written in-house^78^. The region corresponding to water (4.66–5.0 ppm) was removed from the spectra, and the data were imported into SIMCA-P software (Umetrics) for PLS-DA. The heat map was constructed with a relative *Z*-score of peak area of each metabolite identified by NMR and LC-MS.

### RNA sequence analysis

RNA was isolated using RNAzol (Molecular Research Center) according to the manufacturer’s instructions. RNA was dissolved in the TE buffer (Thermofisher), and remaining DNA contamination was removed using DNAse I (Sigma-Aldrich) for 30 min. To clean RNA, the same volume of 8 M LiCl (Sigma-Aldrich) was added and incubated overnight at −20 °C. Samples were centrifuged for 30 min at 16,100×*g*/2 °C; the supernatant was removed and the pellet washed twice in 80% ethanol, followed by centrifugation as above. The remaining ethanol was evaporated at 65 °C for 10 min, and RNA was dissolved in 20 µl of TE buffer. Total RNA concentration was measured by Nanodrop 2000, and its quality was assessed by the Fragment Analyzer (Advanced Analytical) using Standard Sensitivity RNA Analysis Kit (Advanced Analytical). The libraries were prepared from 2 µg of total RNA using QuantSeq 3’mRNA-Seq Library Prep Kit FWD for Illumina (Lexogen), pooled and sequenced on MiSeq v3 kit in the 150 bp SE mode.

About 2 million reads per sample were obtained, and low-quality reads were filtered out using TrimmomaticSE (v. 0.36^79^) with parameters “trimmomatic/TRIM_MDA_K4_1_sequence.fastq CROP:165 HEADCROP:12 ILLUMINACLIP:~/Lexogen_quantseq.fa:2:30:10 LEADING:3 TRAILING:3 SLIDINGWINDOW:4:15 MINLEN:36”. Ribosomal and mitochondrial RNA reads were removed using Sortmerna (v 2.1b^80^) using default parameters. The remaining reads were aligned to the Human Genome Version GRCh38.87 and count tables were generated using STAR (v 2.5.2b^81,82^). Differentially expressed genes (DEGs) were analyzed by means of DESeq2 (v 1.18.0,^82^) using the default function “DESeq” without altered parameters. Result tables were generated as contrast between the SDHB^KO^ and parental, SDHB^KO^SDHA^low^ and parental, and SDHB^KO^ and SDHB^KO^SDHA^low^ cells. Genes with padj <0.1 for at least one pair of comparisons were chosen as DEGs. The function “plotPCA” from DESeq2 package with default parameters was used for PCA analysis. The heat-map was created from rlog transformed count data after subtraction of their mean values for every DEG. Altogether, we identified 523 significantly changed genes out of the total number of 7587 detected genes (DESeq2 BaseMean >5). The data have been deposited in NCBI’s Gene Expression Omnibus and are accessible through GEO Series accession number GSE108938.

For gene set enrichment analysis, DEGs were filtered and used for clustering and ontology analysis. The normalized counts of these genes for all biological replicates and cell lines (parental–MDA231, SDHB^KO^–SHB, and SDHB^KO^SDHA^low^–SH1) were used to analyze for patterns of expression across the three cell lines. The data were first normalized across cell lines by dividing the mean of the replicates by the sum of the mean of the replicates. The R package optCluster (R ver. 3.4.3) was then used to determine the best clustering algorithm among agnes, clara, diana, hierarchical, kmeans, pam, and sota clustering methods using the validation methods connectivity, Dunn index and silhouette width, and the number of clusters set to ten^83^. Clusters that showed similar expression profiles were then merged.

Gene ontology was performed on the genes from each unique cluster, using the R package gprofiler, to identify significantly (*p*-value < 0.05) enriched GO terms^84^. The genes were analyzed with no specified ranking while the gSCS (Set Counts and Sizes) algorithm was used to determine the significance threshold. All identified genes from the RNASeq were used as background. GO annotations inferred by in silico methods were included in the analysis.

After the genes were linked to their appropriate GO terms, REVIGO was used to cluster similar GO terms together while using the *p*-value output from gprofiler to create a ranked list^85^. A similarity of 0.7 using the SimRel semantic similarity method was used to group similar GO terms and the UniProt database (15 March 2017) was used to find the relevant GO term sizes. Output from REVIGO was visualized as treemaps.

### 2D in-cell NMR analysis

Six plates of 70% confluent cultured cells were harvested with centrifugation. After re-suspending the cells with 5 ml DPBS, the cells were counted and 3 × 10^7^ cells were transferred into a fresh tube. After centrifugation, the harvested cells were re-suspended in 500 μl glucose-free DMEM media (Gibco) supplemented with 10% dialyzed FBS, 25 mM U-^13^C_6_-labeled glucose, and 10% D_2_O. The cells were spun in an NMR (30 g, 100 s) to allow sedimentation, enough to cover the active region of the NMR detection coil. ^1^H-^13^C Heteronuclear Single Quantum Coherence (HSQC) NMR spectra were measured using the 800-MHz Bruker Avance spectrometer equipped with a cryogenic triple resonance probe (Seoul National University). The dataset comprises 1024 × 128 points for direct and indirect dimensions, respectively. The time course spectral measurement was obtained at 310 K for 24 time points, with each experiment lasting for 288 s (13 points). Each metabolite was identified by spiking the standard compound.

### Transmission electron microscopy

TEM was accomplished as follows. Cells were grown on cover-slips, fixed with 2% glutaraldehyde (Agar Scientific) and post-fixed with 1% OsO_4_ made up in Sorensen’s phosphate buffer (0.1 M, pH 7.2–7.4), dehydrated, and embedded in Epon-Durkupan (Sigma-Aldrich). Ultrathin sections (~70–90 nm) were cut, contrasted with uranyl acetate (Agar Scientific), and examined in the Morgagni 268 transmission electron microscope (FEI) at 80 kV and in the TECNAI G2 20 LaB6 electron microscope (FEI) at 200 kV. Images were captured with Mega View III CCD camera (Olympus Soft Imaging Solutions).

### Paraganglioma patients

Tumor tissue from 6 patients (3 with *SDHB* mutations, 1 with *SDHA* mutation, 2 sporadic) was used in this study. Tumor tissue was obtained during surgery at the National Institutes of Health (NIH). Genetic testing was performed by NIH in collaboration with the Mayo Clinic (Rochester, MN, USA) or the results were sent from external facilities to the NIH. Expression of SDHB and SDHA proteins was also evaluated by immunohistochemistry as previously described^86^. Signed written consent was obtained from all patients prior to any experimental work. The use of patient samples was allowed by the Eunice Kennedy Shriver NICHD IRB (Ethics Approval #00-CH-0093). The tumor samples examined were as follows (Sample code, affected subunit, DNA base pair change, protein amino acid change): S/ne7, SDHB, c.183T>G, p.Tyr61X; S-275, SDHB, c.526G>T, p.Glu17X; S-73, SDHB, c.268C>T, p.Arg90X; S-281 A/B, SDHA, c.91C>T, p.Arg31X; S-187, Sporadic, no CII subunit affected; S-76, Sporadic, no CII subunit affected.

### Statistical evaluation

Unless stated otherwise, data are mean values ± SD of at least three independent experiments. In mouse experiments, groups of 6 animals were used, unless stated otherwise. The two-tailed unpaired Student’s *t*-test was used to assess statistical significance with *p* < 0.05 being regarded as significant. Images are representative of three independent experiments.

### Data availability

RNA sequence data are filed in NCBI’s Gene Expression Omnibus and are accessible through GEO Series accession number GSE108938. The mass spectrometry proteomics data have been deposited to the ProteomeXchange Consortium via the PRIDE partner repository with the dataset identifier PXD009656. All other data that support findings of this study are available from the corresponding authors upon request.

## Electronic supplementary material

Supplementary Information

Description of Additional Supplementary Files

Supplementary Data 1

Supplementary Data 2

Supplementary Data 3
