# Supplementary material for: Alternative assembly of respiratory complex II connects energy stress to metabolic checkpoints
Source: Nat Commun. 2018 Jun 7;9:2221. doi: 10.1038/s41467-018-04603-z (PMC5992162; doi:10.1038/s41467-018-04603-z)
Supplement: Supplementary file 1 — Supplementary Information [file 41467_2018_4603_MOESM1_ESM.pdf]

Alternative assembly of respiratory complex II connects energy stress to metabolic checkpoints

Bezawork-Geleta et al.

Supplementary Information

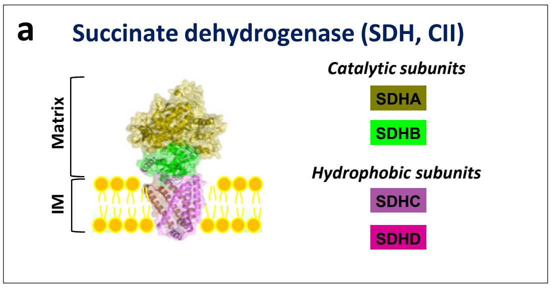

b

Cell lines	Genetic modification	SDHA expression (%)	SDHB expression (%)	SDHC expression (%)	SDHD expression (%)
Parental	-	100	100	100	100
SDHB^{KO}	TALEN	100	nil	nil	n.d.*
SDHB^{KO} EV	TALEN (Empty Vector)	100	nil	nil	n.d.
SDHB^{KO} SDHA^{low}-1	TALEN RNAi/shRNA	25	nil	nil	n.d.
SDHB^{KO} SDHA^{low}-2	TALEN RNAi/shRNA	35	nil	nil	n.d.

*n.d., not determined experimentally, since there is no available anti-SDHD IgG; It is expected that the level of SDHD is similar to that of SDHC.

Supplementary Figure 1 Crystal structure showing assembly of CII subunits and their steady state level in MDA231 sublines. **a** Crystal structure of fully assembled CII as determined from porcine mitochondria PDB:1ZOY. **b** Gene editing and RNAi techniques used to generate MDA231 sublines.

a

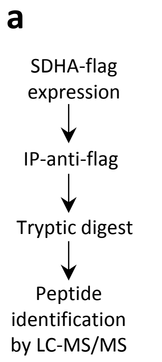

N	Score	%Cov(95)	Accession	Protein ID	Name	Peptides(95%)
1	26.21	21.74	P04264	K2C1_HUMAN	Keratin, type II cytoskeletal 1	13
2	15.04	12.67	P13645	K1C10_HUMAN	Keratin, type I cytoskeletal 10	7
3	11.07	10.59	P35527	K1C9_HUMAN	Keratin, type I cytoskeletal 9	6
4	10.63	31.93	Q9NX18	SDHF2_HUMAN	Succinate dehydrogenase assembly factor 2, mitochondrial	6
5	7.72	8.764	P35908	K22E_HUMAN	Keratin, type II cytoskeletal 2 epidermal	5
6	5.9	22.02	Q9GZT3	SLIRP_HUMAN	SRA stem-loop-interacting RNA-binding protein, mitochondrial	2
7	5.05	23.65	Q04837	SSBP_HUMAN	Single-stranded DNA-binding protein, mitochondrial	3
8	4.46	16.95	P62316	SMD2_HUMAN	Small nuclear ribonucleoprotein Sm D2	3
9	4.09	24.07	Q5VUM1	SDHF4_HUMAN	Succinate dehydrogenase assembly factor 4, mitochondrial	2
10	4	15.2	P62899	RL31_HUMAN	60S ribosomal protein L31	2
11	3.92	13.1	P39019	RS19_HUMAN	40S ribosomal protein S19	2
12	3.66	15.08	P62318	SMD3_HUMAN	Small nuclear ribonucleoprotein Sm D3	2
13	2.82	8.097	P07477	TRY1_HUMAN	Trypsin-1	5
14	2.25	10.43	P05387	RLA2_HUMAN	60S acidic ribosomal protein P2	1
15	2.03	7.143	P62829	RL23_HUMAN	60S ribosomal protein L23	1
16	2	11.11	P06309	KV205_HUMAN	Ig kappa chain V-II region GM607 (Fragment)	1
17	2	8.462	Q9Y2R5	RT17_HUMAN	28S ribosomal protein S17, mitochondrial	1
18	2	7.246	Q6P161	RM54_HUMAN	39S ribosomal protein L54, mitochondrial	1
19	2	8.966	Q15388	TOM20_HUMAN	Mitochondrial import receptor subunit TOM20 homolog	1
20	2	16.81	P62314	SMD1_HUMAN	Small nuclear ribonucleoprotein Sm D1	1
21	2	2.463	P02768	ALBU_HUMAN	Serum albumin	1
22	1.57	6.618	P61353	RL27_HUMAN	60S ribosomal protein L27	1
23	1.51	6.977	Q9BTM1	H2AJ_HUMAN	Histone H2A.J	1
24	1.33	5.97	P82664	RT10_HUMAN	28S ribosomal protein S10, mitochondrial	1
25	1.26	4.79	Q96EL2	RT24_HUMAN	28S ribosomal protein S24, mitochondrial	1
26	0.97	5.797	O15235	RT12_HUMAN	28S ribosomal protein S12, mitochondrial	1
27	0.92	8.491	Q9Y291	RT33_HUMAN	28S ribosomal protein S33, mitochondrial	1

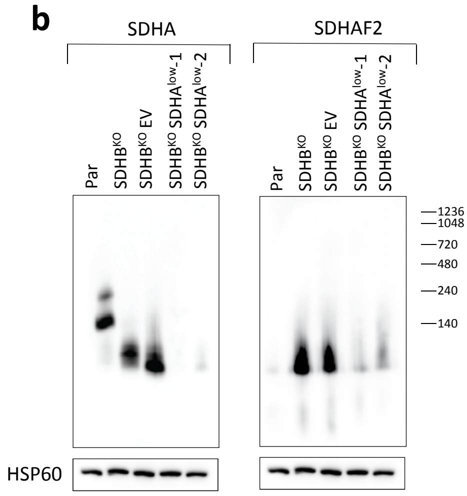

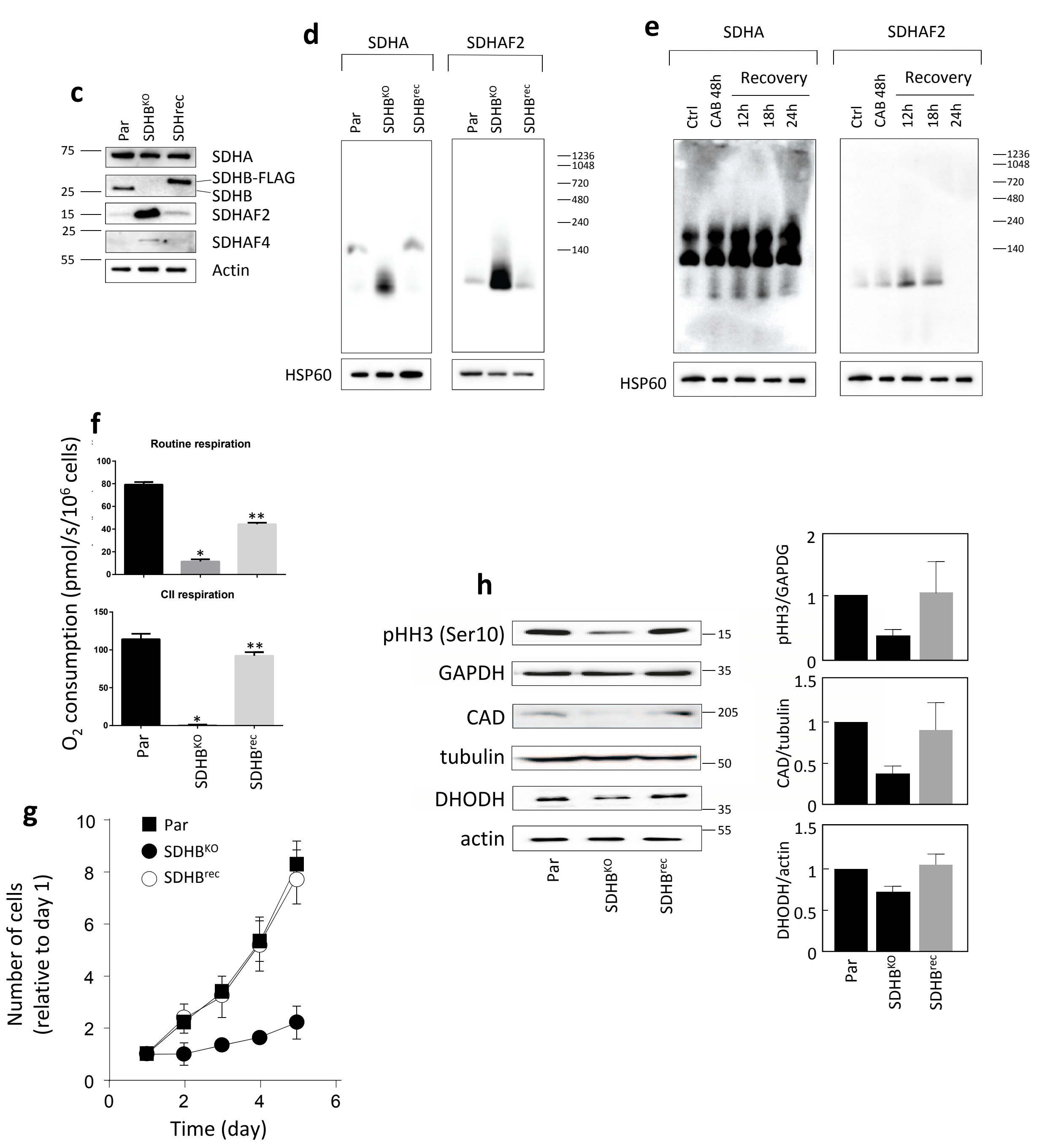

Supplementary Figure 2 SDHB^{KO} cells transfected with SDHA-FLAG were processed as indicated in the flow chart on the left, and the proteins identified by MS are shown in the table on the right (**a**). **b** Cells were subjected to NBGE followed by WB for SDHA and SDHAF2. Parental, SDHB^{KO} and SDHB^{KO} cells expressing SDHB-FLAG (SDHB^{rec} cells) were subjected to SDS-PAGE and assessed for the level of SDHA, SDHB, SDHAF2 and SDHAF4 by WB (**c**) and analyzed by NBGE followed by WB for the level of SDHA and SDHAF2 (**d**). **e** Parental cells were exposed to CAB for 48 h, allowed to recover for the time periods shown, and assessed for the level of SDHA and SDHAF2 using NBGE followed by WB. The data shown comes from the same mitochondrial preparation, the same HSP60 control therefore applies to both panels. Parental, SDHB^{KO} and SDHE^{rec} cells were assessed for routine and CII-dependent oxygen consumption (**f**), proliferation in galactose- and glucose-containing media (n=4) (**g**), and for the level of pHH(Ser10), CAD and DHODH using SDS-PAGE and WB (**h**). Images are representative of three independent experiments. Data in panel **f** are mean values ± S.D. The symbol * indicates significant differences compared to parental cells with $p<0.05$ and ** significant differences compared to SDHB^{KO} cells with $p<0.05$., One-way ANOVA, GraphPad Prism 6.0, n = 4.

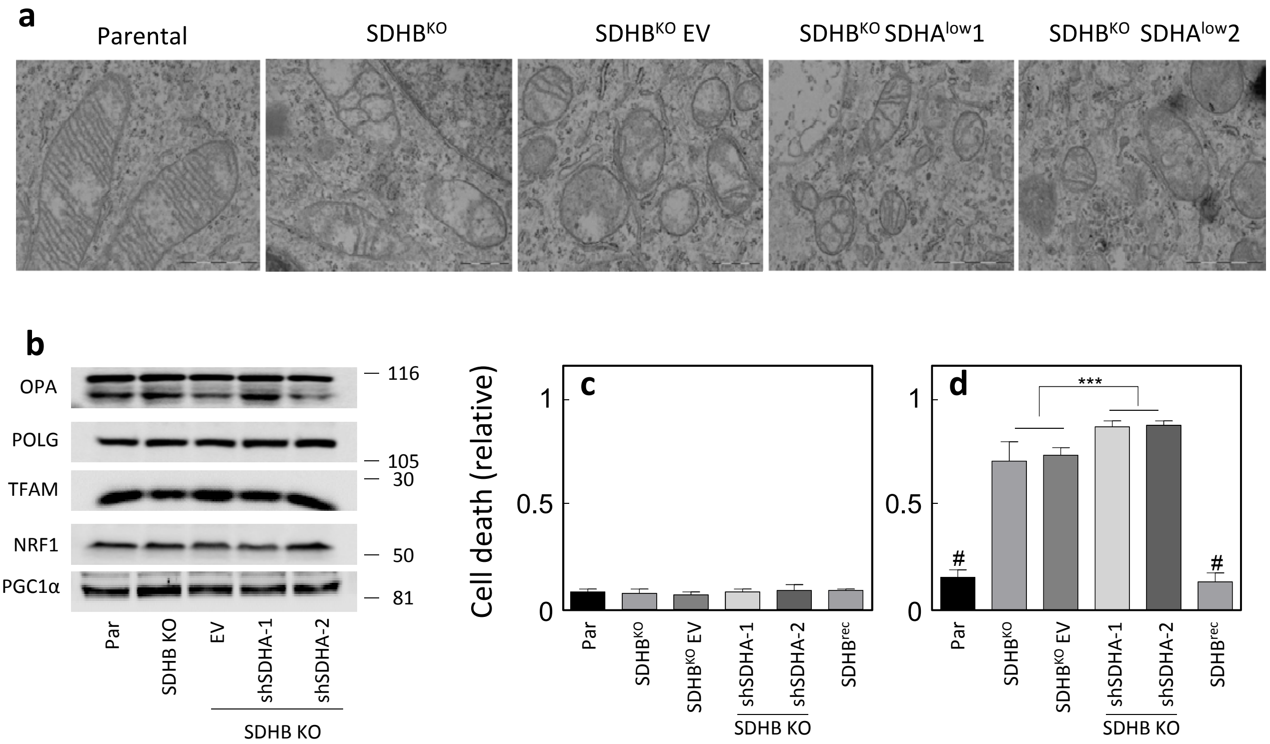

Supplementary Figure 3 **a** Cells were evaluated for mitochondrial morphology using TEM (scale bar, 1 μm) and for the level of expression of OPA, POLG, TFAM, NRF1 and PGC1α using SDS-PAGE/WB (**b**). Cell lines, as indicated, were maintained in glucose- (**c**) and galactose-containing media (**d**) and assessed for cells death using standard annexin V/PI assay. Images in panels (**a**) and (**b**) are representative of three independent experiments, data in panels (**c**) and (**d**) are mean values ± S.D. (n=4), with the symbol *** indicating significant difference between $SDHB^{KO}$ and $SDHB^{KO}$ EV cells versus $SDHB^{KO}$ $SDHA^{low}$ cells with $p<0.05$, the symbol # significant difference between $SDHB^{KO}$, $SDHB^{KO}$ EV and $SDHB^{KO}$ $SDHA^{low}$ cells versus parental and $SDHB^{rec}$ cells with $p<0.0001$., one-way ANOVA, GraphPad Prism 6.0.

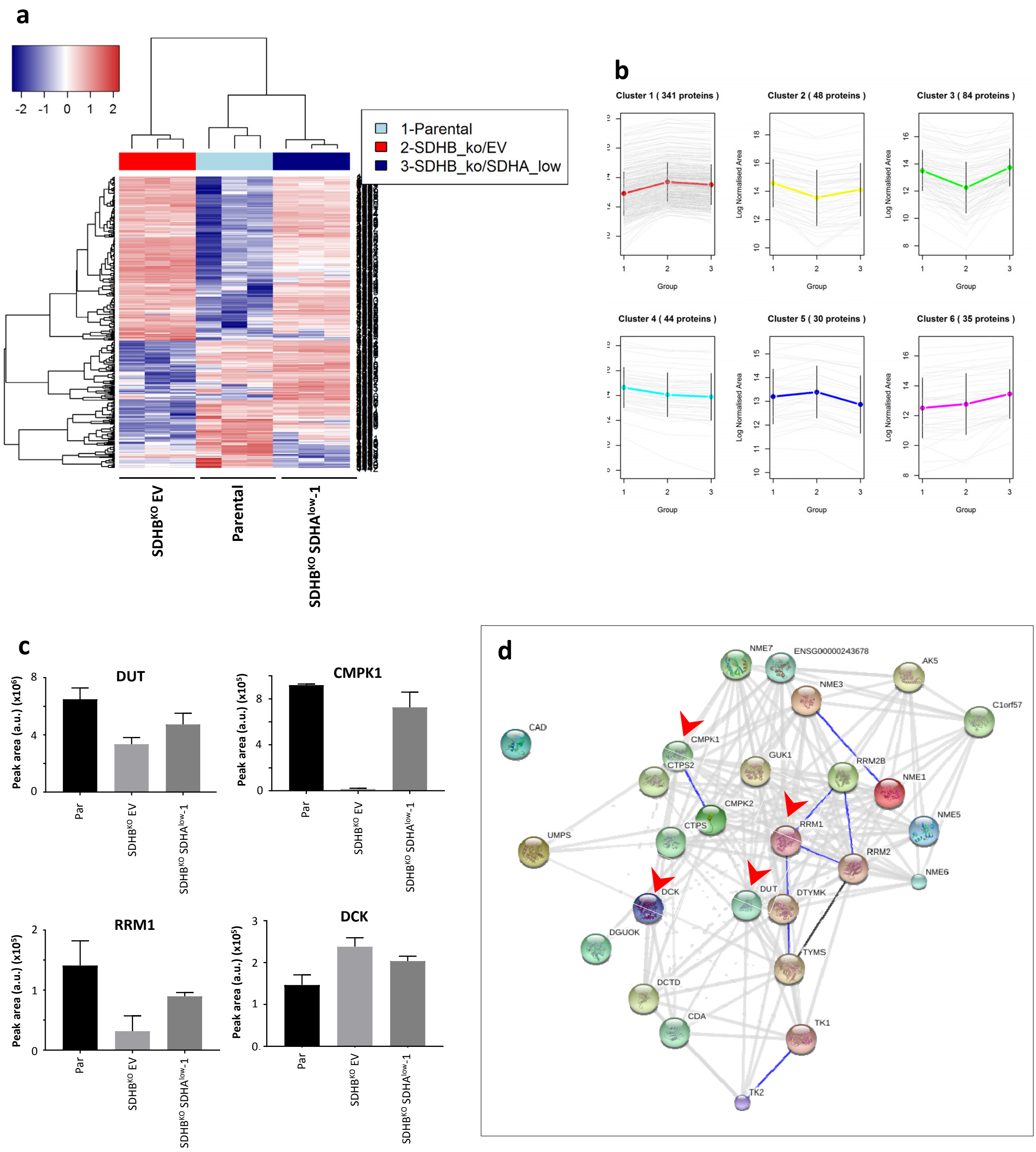
a
-2 -1 0 1 2
1-Parental
2-SDHB_ko/EV
3-SDHB_ko/SDHA_low
SDHBKO EV
Parental
SDHBKO SDHAlow-1
b
Cluster 1 (341 proteins)
Cluster 2 (48 proteins)
Cluster 3 (84 proteins)
Cluster 4 (44 proteins)
Cluster 5 (30 proteins)
Cluster 6 (35 proteins)
Log Normalised Area
Group
c
DUT
CMPK1
RRM1
DCK
Peak area (a.u.) (x10⁶)
Peak area (a.u.) (x10⁵)
Par
SDHBKO EV
SDHBKO SDHAlow-1
d
NME7
ENSG00000243678
AK5
NME3
C1orf57
CAD
CMPK1
CTPS2
GUK1
RRM2B
NME1
CMPK2
RRM1
NME5
UMPS
CTPS
RRM2
NME6
DCK
DUT
DTYMK
DGUOK
TYMS
DCTD
CDA
TK1
TK2

Supplementary Figure 4 Effects of change in SDHA assembly in proteome of MDA231 sublines. **a** Unsupervised hierarchical clustering of protein expression data from SWATH-MS analysis for indicated sublines. **b** Cluster of proteins with distinct differential expression among MDA231 sublines. Dots connected by solid lines indicate median of log transformed normalized peak area that indicate the steady state of the protein. Vertical lines indicate the corresponding interquartile range. Gray lines mark the individual protein steady state in the cluster. **c** Peak area of several peptides identified by SWATH-MS analysis for most differentially expressed proteins that are involved in the *de novo* pyrimidine pathway. DUT, deoxyuridine 5'-triphosphate nucleotidohydrolase; CMPK1, cytidine/uridine monophosphate kinase 1; RRM1, ribonucleoside-diphosphate reductase large subunit; negative regulator DCK, deoxycytidine kinase. Error bars represent SD, n=3. Those proteins are highlighted with red arrows on a complete picture of STRING interaction network for pyrimidine deoxyribonucleotide *de novo* biosynthesis (accessed from Pathcard) in panel **d**.

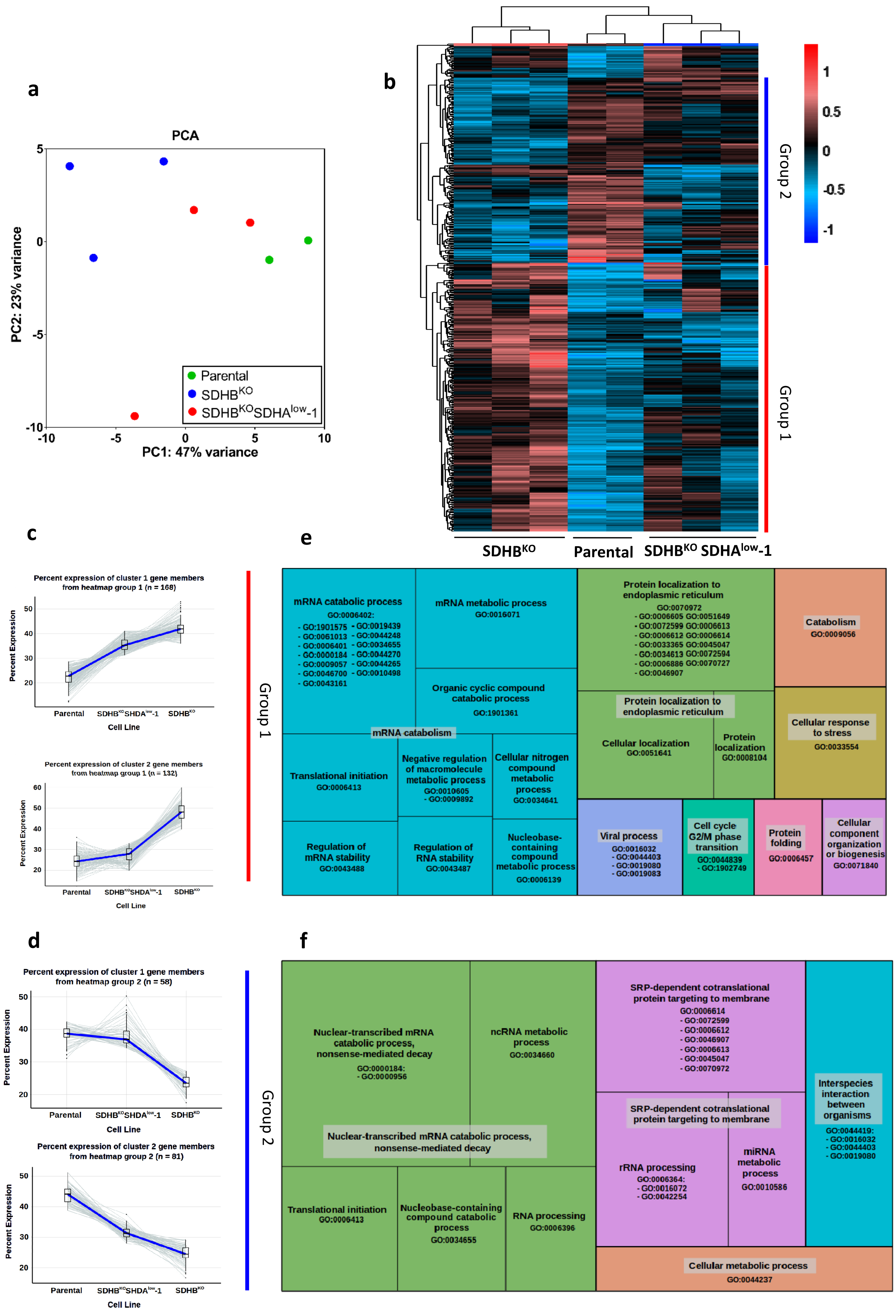

a
PCA
PC1: 47% variance
PC2: 23% variance
Parental
SDHB^KO
SDHB^KO SDHA^low-1
b
Group 2
Group 1
SDHB^KO
Parental
SDHB^KO SDHA^low-1
c
Percent expression of cluster 1 gene members from heatmap group 1 (n = 168)
Percent expression of cluster 2 gene members from heatmap group 1 (n = 132)
Percent Expression
Parental
SDHB^KO SHDA^low-1
SDHB^KO
Cell Line
d
Percent expression of cluster 1 gene members from heatmap group 2 (n = 58)
Percent expression of cluster 2 gene members from heatmap group 2 (n = 81)
e
Group 1
mRNA catabolic process
GO:0006402:
- GO:1901575 - GO:0019439
- GO:0061013 - GO:0044248
- GO:0006401 - GO:0034655
- GO:0000184 - GO:0044270
- GO:0009057 - GO:0044265
- GO:0046700 - GO:0010498
- GO:0043161
mRNA metabolic process
GO:0016071
Organic cyclic compound catabolic process
GO:1901361
mRNA catabolism
Translational initiation
GO:0006413
Negative regulation of macromolecule metabolic process
GO:0010605
- GO:0009892
Cellular nitrogen compound metabolic process
GO:0034641
Regulation of mRNA stability
GO:0043488
Regulation of RNA stability
GO:0043487
Nucleobase-containing compound metabolic process
GO:0006139
Protein localization to endoplasmic reticulum
GO:0070972
- GO:0006605 GO:0051649
- GO:0072599 GO:0006613
- GO:0006612 GO:0006614
- GO:0033365 GO:0045047
- GO:0034613 GO:0072594
- GO:0006886 GO:0070727
- GO:0046907
Protein localization to endoplasmic reticulum
Cellular localization
GO:0051641
Protein localization
GO:0008104
Catabolism
GO:0009056
Cellular response to stress
GO:0033554
Viral process
GO:0016032
- GO:0044403
- GO:0019080
- GO:0019083
Cell cycle G2/M phase transition
GO:0044839
- GO:1902749
Protein folding
GO:0006457
Cellular component organization or biogenesis
GO:0071840
f
Group 2
Nuclear-transcribed mRNA catabolic process, nonsense-mediated decay
GO:0000184:
- GO:0000956
ncRNA metabolic process
GO:0034660
Nuclear-transcribed mRNA catabolic process, nonsense-mediated decay
Translational initiation
GO:0006413
Nucleobase-containing compound catabolic process
GO:0034655
RNA processing
GO:0006396
SRP-dependent cotranslational protein targeting to membrane
GO:0006614
- GO:0072599
- GO:0006612
- GO:0046907
- GO:0006613
- GO:0045047
- GO:0070972
SRP-dependent cotranslational protein targeting to membrane
rRNA processing
GO:0006364:
- GO:0016072
- GO:0042254
miRNA metabolic process
GO:0010586
Interspecies interaction between organisms
GO:0044419:
- GO:0016032
- GO:0044403
- GO:0019080
Cellular metabolic process
GO:0044237

Supplementary Figure 5. RNA sequence analysis of parental, SDHB^{KO} and SDHB^{KO} SDHA^{low} cells. **a** Principle component analysis of parental, SDHB^{KO} and SDHB^{KO}SDHA^{low} cells. **b** Heatmap of gene expression for genes differentially expressed between the tested cell lines. Direction and fold change in expression compared to the average across all cell lines are color-coded as indicated. The groups of genes used for the gene set enrichment analysis are highlighted in the right margin. **c, d** Clusters of differentially expressed genes that showed (**c**) lower or (**d**) higher transcript abundance in the parental and SDHB^{KO}SDHA^{low}-1 cell lines relative to the SDHB^{KO} cell line (i.e. transcripts the expression of which was reverted back towards the parental cell levels by SDHA knockdown in SDHB^{KO} cells). The trend line is based on the median of the expression value of all genes for a given cell line. Clusters in (**c**) are contained in group 1 in the heatmap, and clusters from (**d**) in group 2. **e, f** Treemaps showing the significant biological processes involving gene members of (**e**) group 1 from the heatmap, or (**f**) group 2 from the heatmap. The major representative and sub-representative GO terms comprising each category are indicated. Each rectangle of the treemap represents a cluster of GO terms involved in similar biological processes. The size of the rectangle is reflective of the p-value significance of the biological process. The boxes of similar color share similar overall processes, while those of different color are more distant.

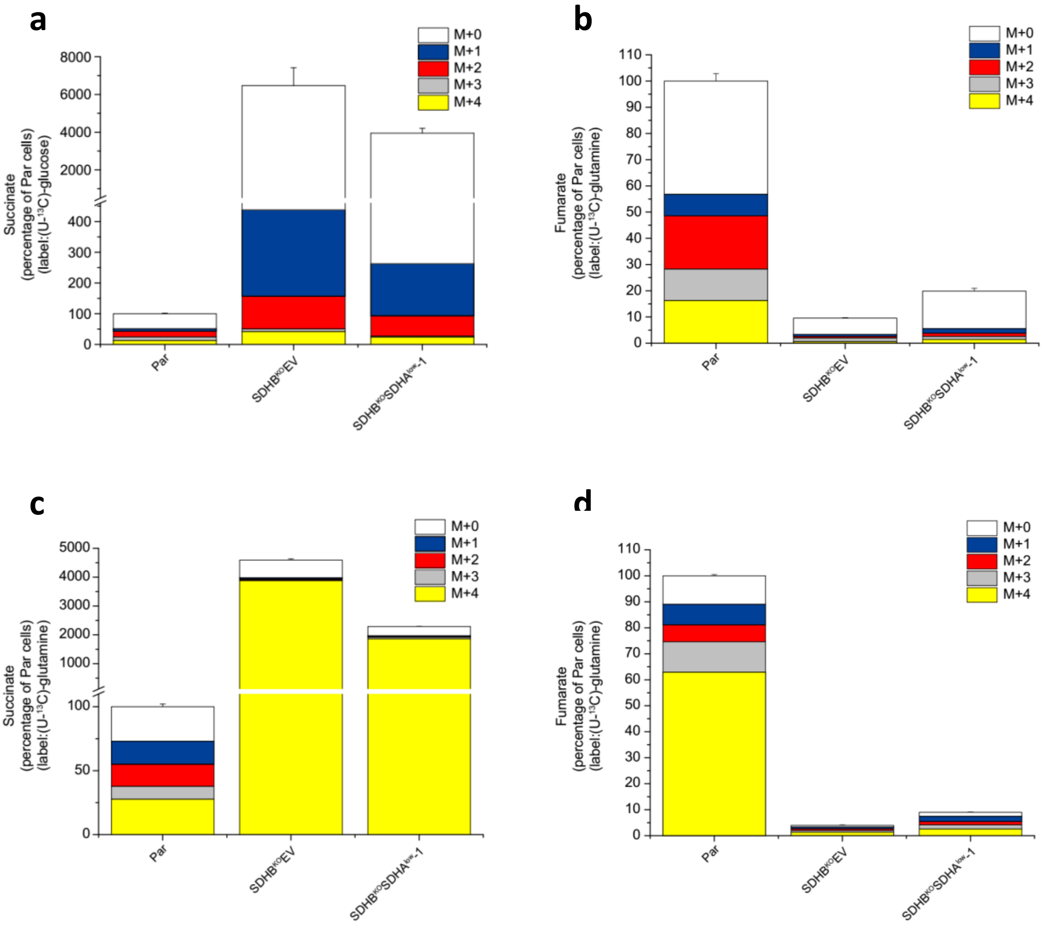

Supplementary Figure 6 Isotopologue distribution of intracellular succinate (**a**, **c**) and fumarate **(b, d)** after incubation for 24 h with 5 mM unlabeled glucose and 20 mM U-^{13}C-glucose (**a**, **b**) or 4 mM U-^{13}C-glutamine (c, d), respectively. All data are presented as mean ± S.D., n=3.

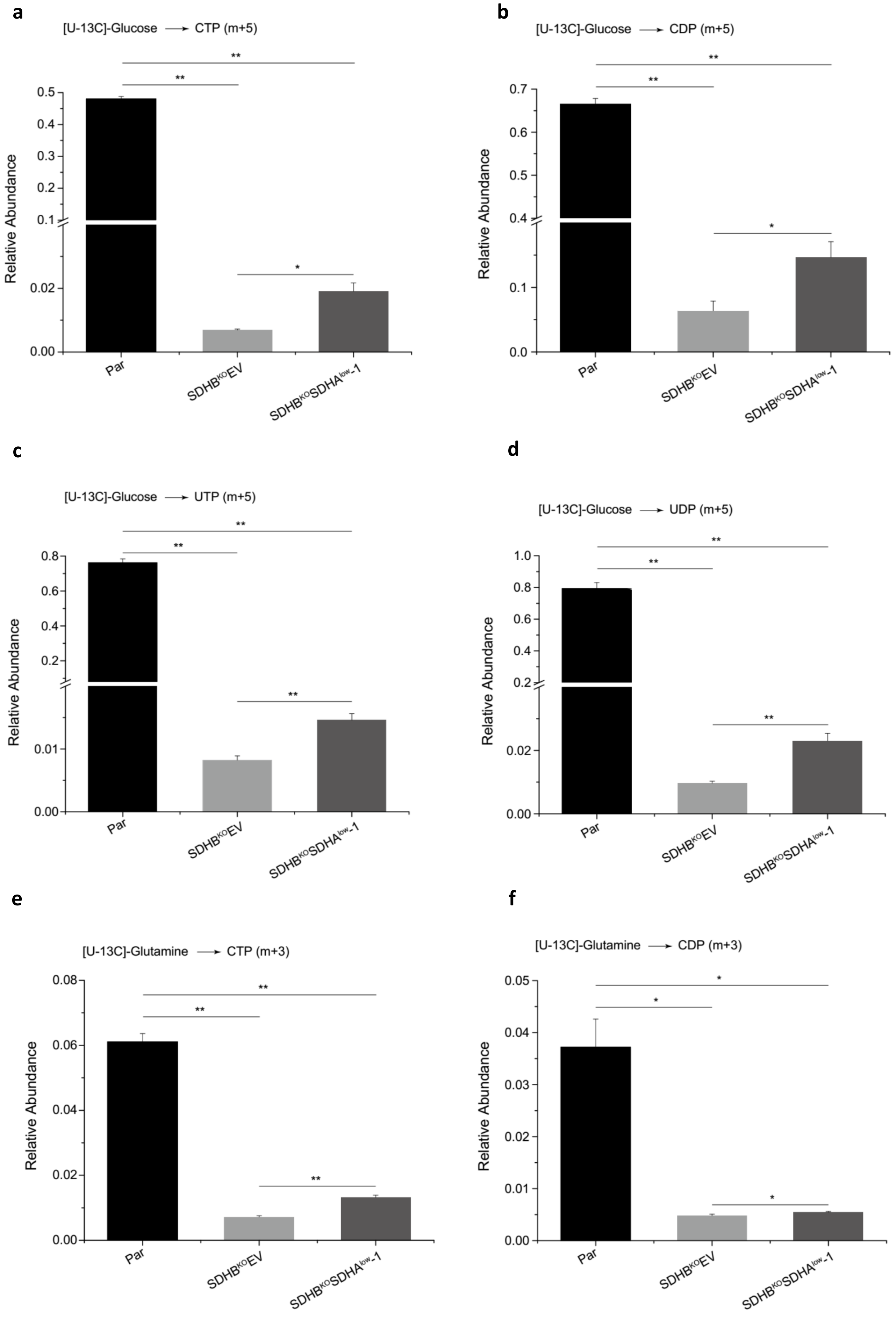
a
[U-13C]-Glucose → CTP (m+5)
**
**
*
0.5
0.4
0.3
0.2
0.1
0.02
0.00
Relative Abundance
Par
SDHB^{KO}EV
SDHB^{KO}SDHA^{low}-1
b
[U-13C]-Glucose → CDP (m+5)
**
**
*
0.7
0.6
0.5
0.4
0.1
0.0
Relative Abundance
Par
SDHB^{KO}EV
SDHB^{KO}SDHA^{low}-1
c
[U-13C]-Glucose → UTP (m+5)
**
**
**
0.8
0.6
0.4
0.2
0.01
0.00
Relative Abundance
Par
SDHB^{KO}EV
SDHB^{KO}SDHA^{low}-1
d
[U-13C]-Glucose → UDP (m+5)
**
**
**
1.0
0.8
0.6
0.4
0.2
0.02
0.00
Relative Abundance
Par
SDHB^{KO}EV
SDHB^{KO}SDHA^{low}-1
e
[U-13C]-Glutamine → CTP (m+3)
**
**
**
0.08
0.06
0.04
0.02
0.00
Relative Abundance
Par
SDHB^{KO}EV
SDHB^{KO}SDHA^{low}-1
f
[U-13C]-Glutamine → CDP (m+3)
*
*
*
0.05
0.04
0.03
0.02
0.01
0.00
Relative Abundance
Par
SDHB^{KO}EV
SDHB^{KO}SDHA^{low}-1

Supplementary Figure 7. Incorporation of ^{13}C label from glucose or glutamine to pyrimidines. (**a** - **d**) Relative isotopomer amounts (M+5) of CTP, CDP, UTP and UDP were assessed by LC-MS/MS using [U-^{13}C] glucose. (**e** and **f**) Relative isotopomer amounts (M+3) of CTP and CDP were assessed by LC-MS/MS using [U-^{13}C] glutamine as tracers. Data shown are mean values ± SD ($n \geq 3$); The symbol ** indicates differences with $p<0.05$ (unpaired t-test).

Uncropped scans from Figure 1

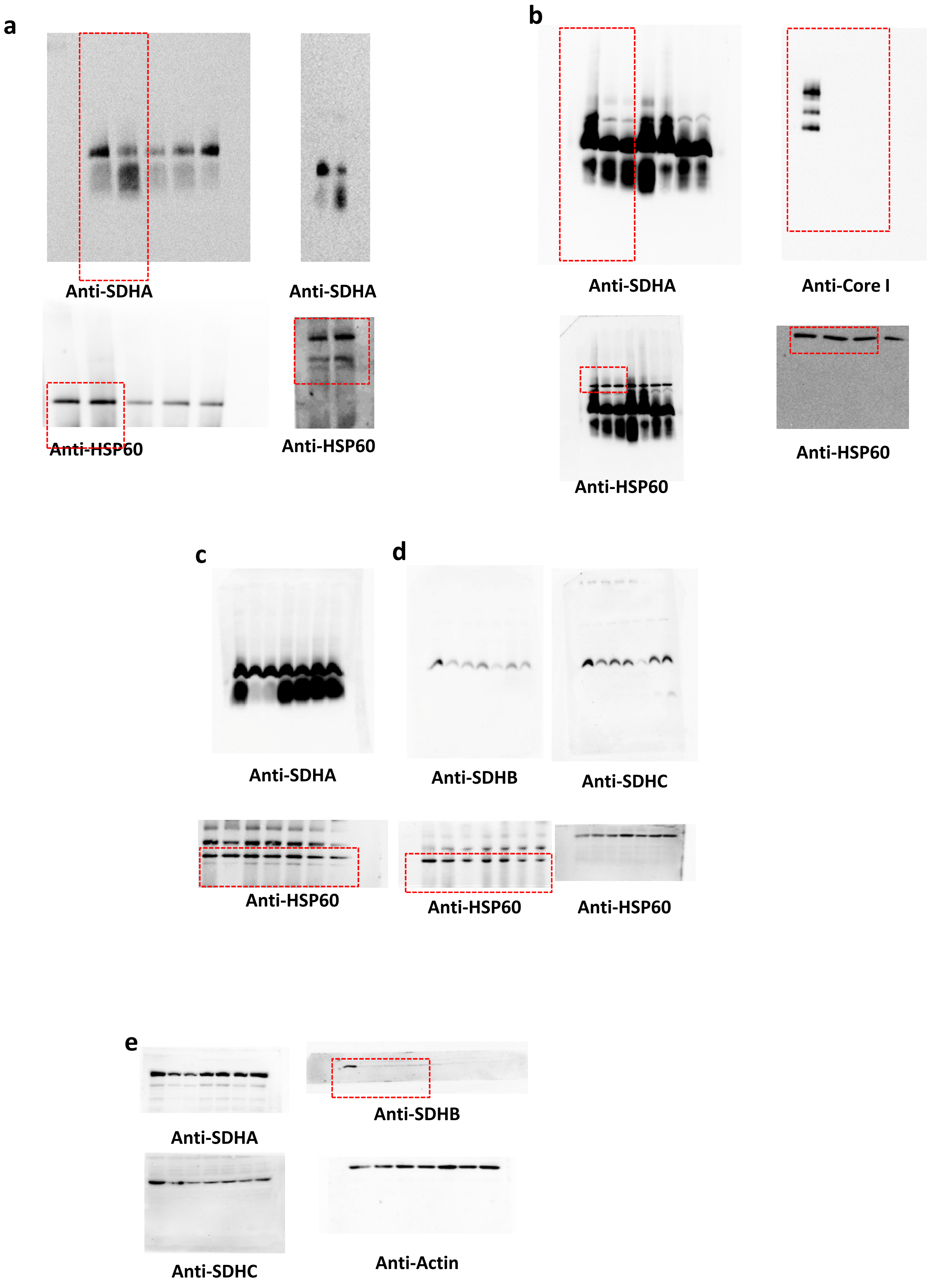

Uncropped scans from Figure 2

c

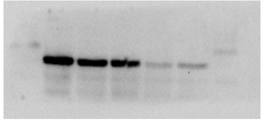

Anti-SDHA

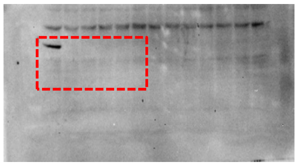

Anti-SDHB

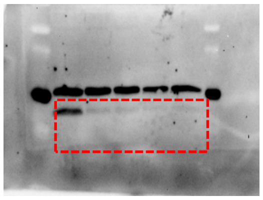

Anti-SDHC

d

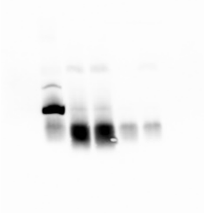

Anti-SDHA

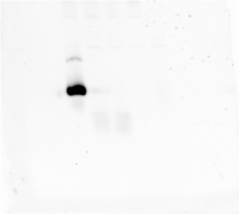

Anti-SDHB

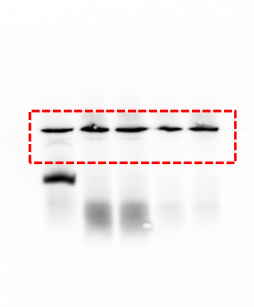

Anti-HSP60

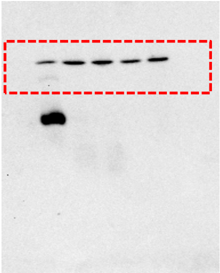

Anti-HSP60

Uncropped scans from Figure 3

g

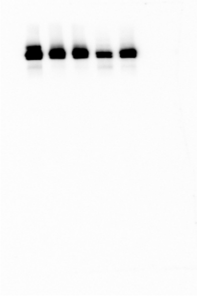

Anti-NDUFA9

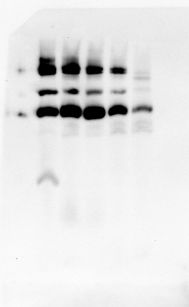

Anti-Core I

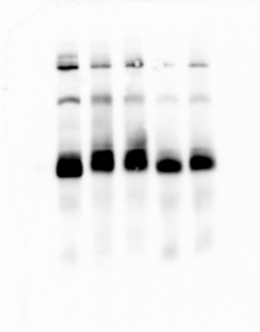

Anti-COXVa

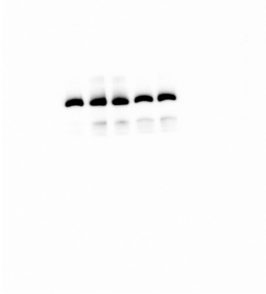

Anti-ATP5

h

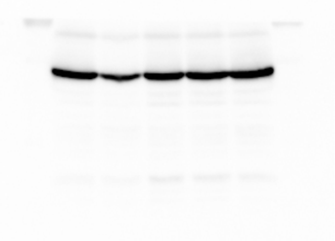

Anti-Core I

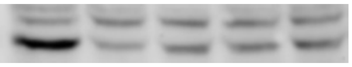

Anti-NDUFV1

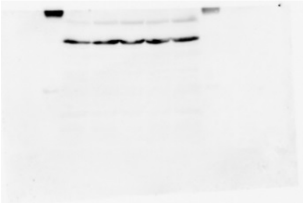

Anti-ATP5

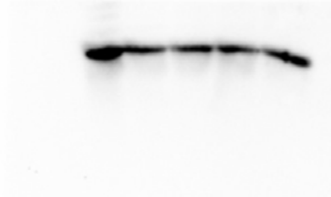

Anti-NDUFS8

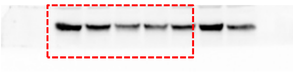

Anti-NDUFA9

Uncropped scans from Figure 4

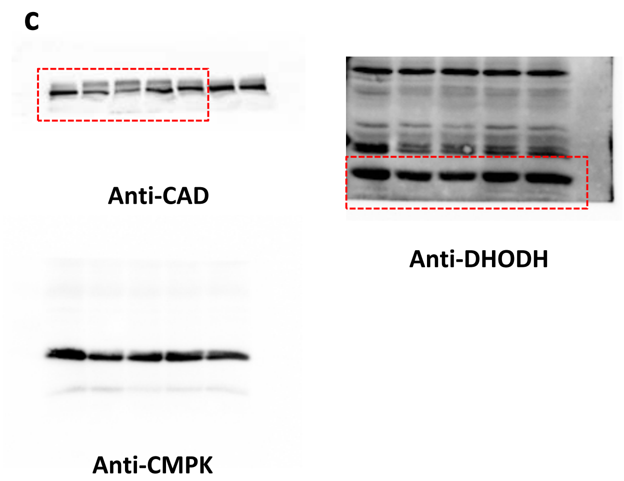

Uncropped scans from Figure 5

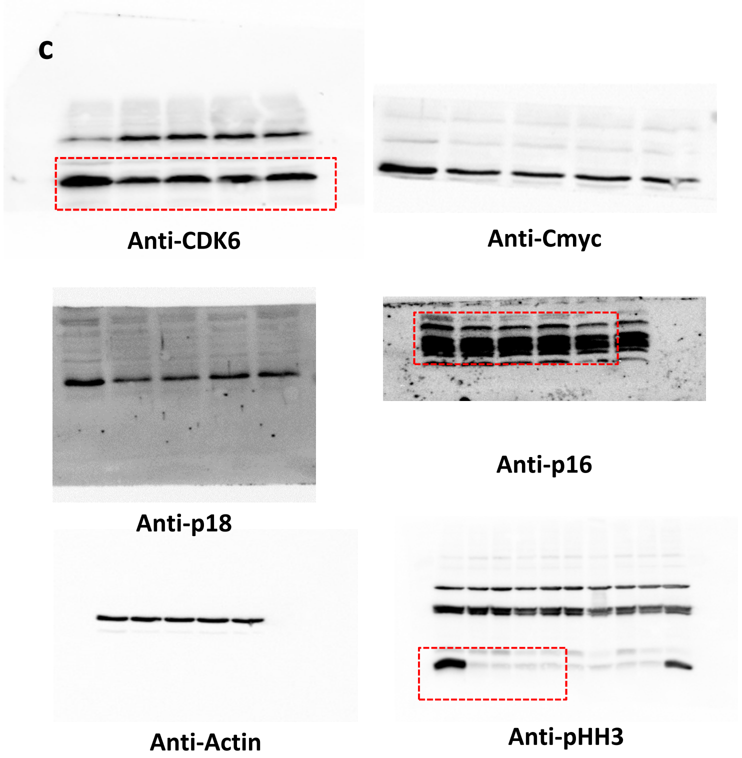

Uncropped scans from Figure 8

b

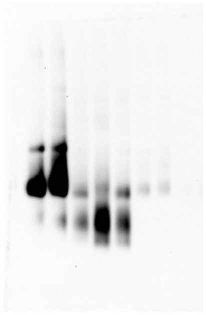

Anti-SDHA

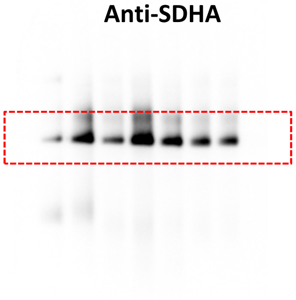

Anti-HSP60

c

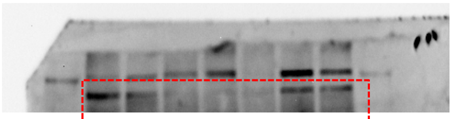

Anti-CAD

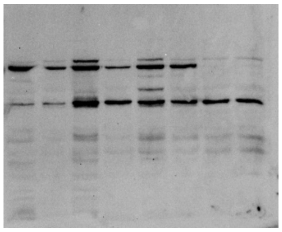

Anti-FAD and Anti-actin

d

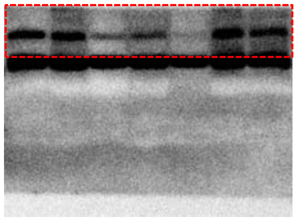

Anti-p18

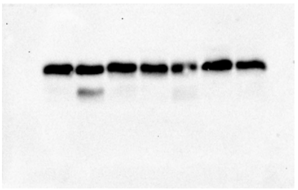

Anti-p21

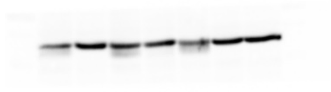

Anti-Actin

Uncropped scans from Supplementary Figure 2

The main figure already contains images of full-length membranes for NBGE

b

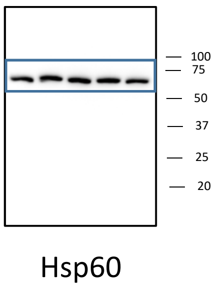

c

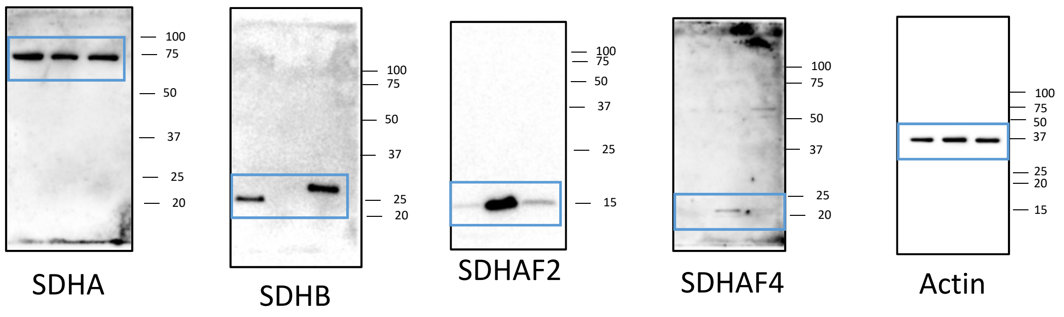

d

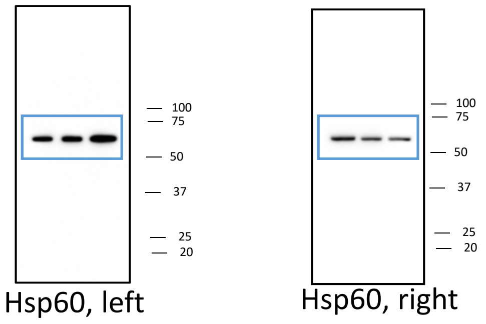

e

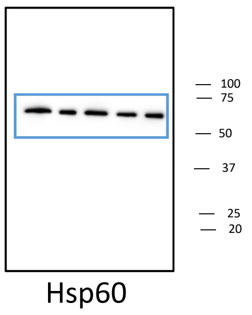

h

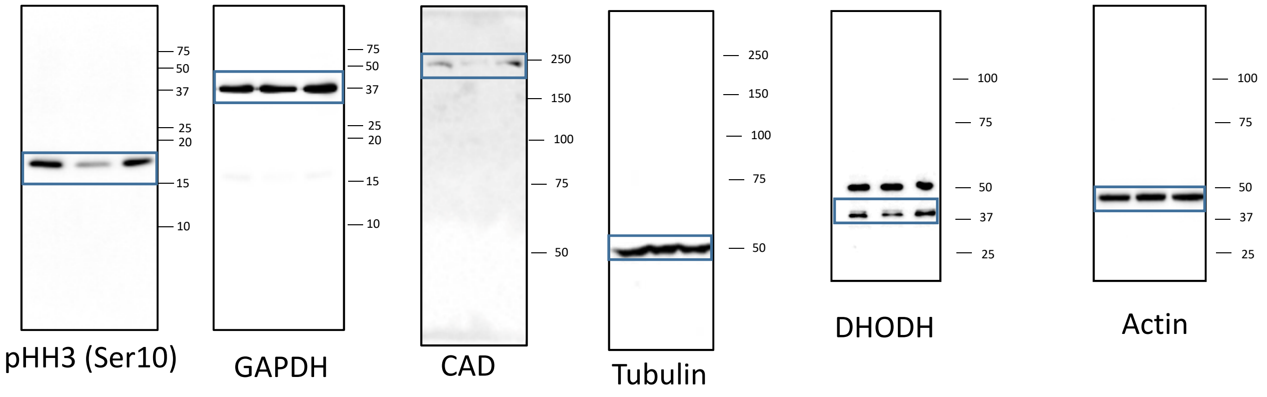

Supplementary Figure 8. Uncropped scans of WB membranes.
