# Supplementary material for: Alternative assembly of respiratory complex II connects energy stress to metabolic checkpoints
Source: Nat Commun. 2018 Jun 7;9:2221. doi: 10.1038/s41467-018-04603-z (PMC5992162; doi:10.1038/s41467-018-04603-z)
Supplement: Supplementary file 2 — Description of Additional Supplementary Files [file 41467_2018_4603_MOESM2_ESM.pdf]

Description of Additional Supplementary Files

File Name: Supplementary Data 1
Description: SDHA-interacting proteins. Complete data from the MS analysis following anti-flag IP for the identification of proteins interacting with SDHA in $CII_{low.}$

File Name: Supplementary Data 2
Description: MS SWATH-detected proteins.

File Name: Supplementary Data 3
Description: Raw data and Z-score for the metabolites identified by NMR and LC-MS/MS from MDA231 sublines. The actual peak intensity of each metabolites and the calculated values of mean and standard deviation (STDEV) are provided. The Z-score was obtained by dividing the difference between actual peak intensities and mean value by the STDEV. Those bolded were identified with LC-MS/MS.
